# The semi-arid ecosystem of Asiatic Lion Landscape in Saurashtra, Gujarat: Population density, biomass and conservation of nine wild prey species

**DOI:** 10.1371/journal.pone.0292048

**Published:** 2023-09-28

**Authors:** Mohan Ram, Aradhana Sahu, Nityanand Srivastava, Rohit Chaudhary, Lahar Jhala, Yashpal Zala

**Affiliations:** 1 Wildlife Division, Sasan-Gir, Junagadh, Gujarat, India; 2 Wildlife Circle, Junagadh, Gujarat, India; 3 Chief Wildlife Warden, Gujarat State, Gandhinagar, Gujarat, India; 4 Department of Wildlife Sciences, Navsari Agricultural University, Navsari, Gujarat, India; Amity University, INDIA

## Abstract

The present study aimed to assess the population density, structure, and population change of nine wild prey species in the semi-arid landscape of Saurashtra, Gujarat, India. A total of eight sites, representing a gradient from highly protected woodlands and grasslands to unreserved grasslands, were selected for sampling. We employed the road transect methodology under a distance sampling framework to achieve our objectives. We evaluated the realized growth rate of the Gir ungulate population through linear regression analysis. Our findings revealed that deer species exhibited higher density and biomass in woodlands compared to grasslands and coastal forests. On the other hand, antelopes showed higher density and biomass in grasslands and coastal forests compared to woodlands. The density gradient of wild prey species was influenced by various factors, including habitat structure, social organization, grouping tendencies, and topography. Over the last four decades, the population of wild prey species in Gir showed minimal changes. Our study provides a comprehensive understanding of wild prey species’ density and biomass patterns at the landscape level. The inclusion of findings from ecologically significant and unique areas, such as coastal forests, further enhances the importance of this study. The implications of this study extend beyond the conservation of wild prey species alone; they also contribute to the conservation of the large carnivore guild in the Saurashtra landscape.

## Introduction

Terrestrial wild ungulates are one of the most threatened groups of mammals, and around 57% of the ~250 ungulate species are currently listed as threatened with extinction (including 14 critically endangered species) [1]. The important reasons for the decline in wild ungulate population are habitat loss, poaching, grazing, disease spread, etc. [2]. Additionally, wild ungulates exhibit remarkable diversity in terms of body size, ranging from mouse deer to elephants [3]. Body size plays an important role in ungulates’ diet and movement patterns. It can have a profound impact on ecosystem functioning at different scales, from large to fine, due to varying dietary requirements, such as browsing or grazing. For example, ungulates with large body sizes are used to feed on more abundant plant material because they need to satisfy their higher absolute energy need [4]. On the contrary, small-sized ungulates need to satisfy their relatively higher energetic demands and hence have to feed upon high plant items such as new shoots, fresh grass, etc. [4]. Therefore, ungulates as a community could affect the structure and function of vegetation where they inhabit. Moreover, ungulates provide essential ecosystem services, such as seed dispersal in forests, which can significantly affect vegetation regeneration patterns [5]. Furthermore, the survival of wild ungulates is directly linked to the conservation of large predators, as they act as important prey for them [6–8]. Therefore, the conservation of wild ungulates could help conserve sympatric species and the entire ecosystem they inhabit.

The conservation of wild ungulates on a global scale is closely linked to establishing protected areas, such as national parks, wildlife sanctuaries, and game reserves [9]. However, many of these protected areas face challenges such as isolation and small size, which make wild ungulates more vulnerable to unpredictable events and reduce their ecological carrying capacities [10, 11]. To address these issues, conserving wild ungulates requires a landscape-level approach incorporating protected areas and the surrounding multi-use landscape matrix. This approach ensures the long-term conservation of these species by maintaining habitat connectivity and providing suitable habitat within the multi-use landscape for adaptable species.

### Top of form

India, the second most populated nation in the world, harbours a stunning range of wild ungulate species [12]. A wide range of protected area (PA) networks, such as national parks, wildlife sanctuaries, conservation reserves, community reserves, and tiger reserves, are earmarked in India for biodiversity conservation, including wild ungulates [13]. However, the PA network comprises around 4% of India’s total geographical area, while 96% is the human use matrix [13]. In such a scenario, landscape-level conservation strategies, as defined above, have been advocated for conserving wild ungulates in India [12]. Nevertheless, developing landscape-level wild ungulate conservation strategies requires robust scientific information on their status, population density, and abundance using widely accepted scientific methodology. Therefore, generating information on population parameters such as density, biomass and age and sex ratio from a multi-use landscape matrix could help formulate conservation strategies for wild ungulates.

**Fig 1:**
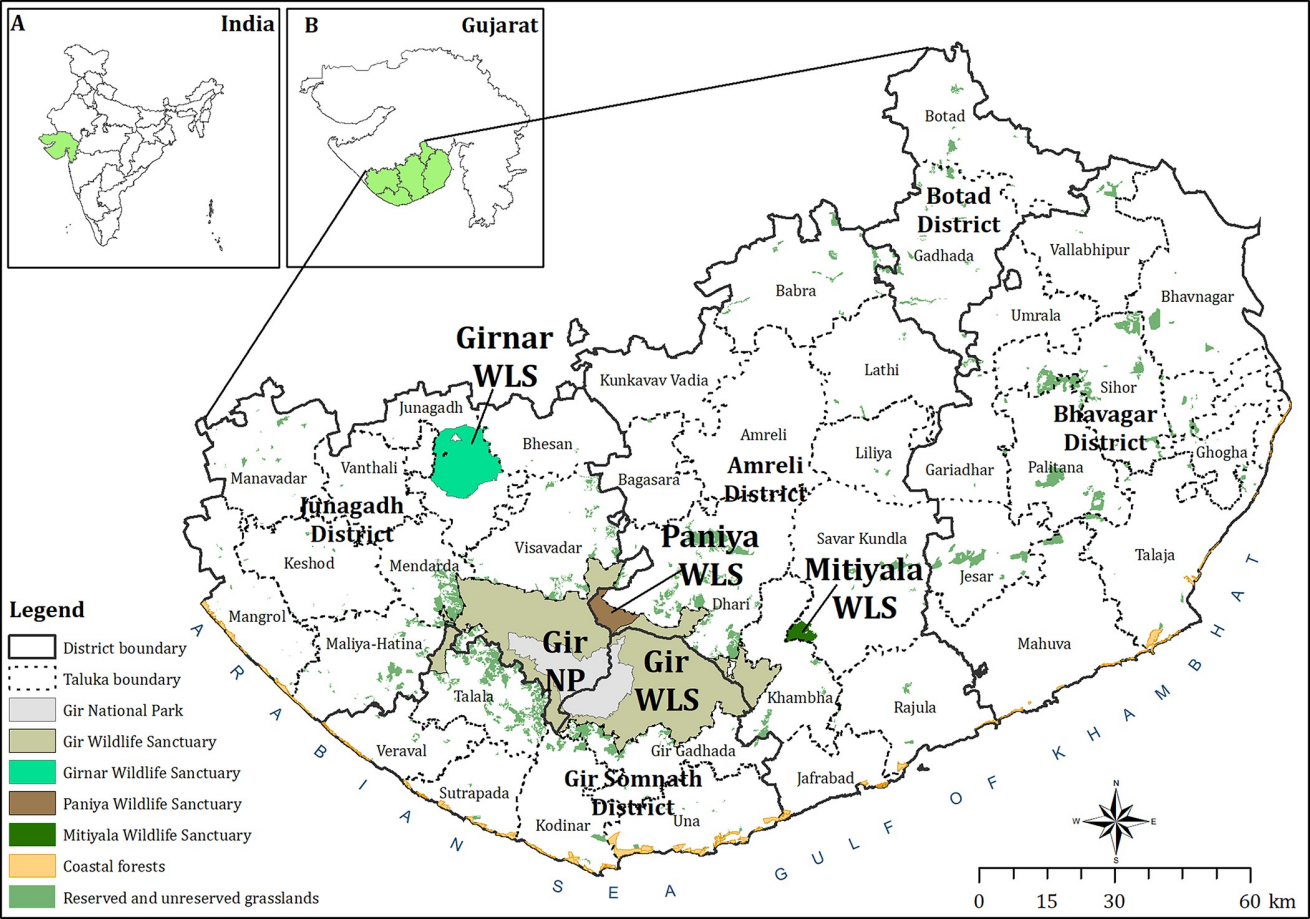
Map of Saurashtra landscape along with eight study sites where prey population was assessed. Map insets indicate the location of Gujarat in India (A) and the study area in Gujarat state (B).

The Saurashtra landscape is situated in the semi-arid biogeographic zone of India and consists of both protected areas and multi-use landscape matrix (Fig 1). Conservation of wild ungulates in the Saurashtra landscape is also linked to the conservation of the endangered Asiatic lion, for which wild ungulates act as principal prey [14]. We carried out a study to understand the status and distribution of nine wild prey species in the Saurashtra landscape, which includes seven wild ungulates (spotted deer *Axis axis*, sambar *Rusa unicolor*, blue bull *Boselaphus tragocamelus*, four-horned antelope *Tetracerus quardicornis*, blackbuck *Antelope cervicapra*, Indian gazelle *Gazella benneti*, wild pig *Sus scrofa*), one primate (hanuman langur *Semnopithecus entellus*) and one large bird (Indian peafowl *Pavo cristatus*). Objectives of the present study were a) To assess the density and biomass of nine wild prey species, 2) To assess the age and sex composition of species under study, and 3) To provide conservation and research implications of the study for wild prey species conservation in the landscape. Due to their strong protection status and minimal human disturbance, we hypothesized that protected areas would harbour a higher density of wild prey species than the habitat patches in the multi-use landscape matrix.

## Materials and methods

### Study area

The study was carried out in the Asiatic Lion Landscape (Fig 1) [15], which is located in the south-western part of the Saurashtra region (20°50’ - 23°5’ N and 69°20’ - 72°10’ E). Flanked by the Gulf of Khambhat and the Arabian Sea, dominated by undulating surfaces broken by hills (Gir, Girnar, Mitiyala, Palitana, etc.) and dissected by various rivers (Shetrunji, Raval, Ardak, Machhundri, Hiran, Shingoda, Shigvada, Ozat, etc.) that flow out in different directions [16, 17], the landscape is a unique mosaic of five protected areas and several small/ large reserved, unreserved, and unclassed forest patches [18].

The landscape characterizes a typical semi-arid biogeographical zone [19] with an aridity index of 20–40% [20]. The mean annual rainfall is ~600 mm [21]. The area’s mean maximum and minimum temperatures are 34°C and 19°C, respectively [22]. The landscape is characterized by three distinct seasons (summer: March-June, monsoon: July-October and winter: November-February). The natural ecosystem in the study area is dominated by agro-pastoral systems and comprises thorn-scrub forests, grasslands, dry deciduous and riverine forests, mangroves, intertidal regions, and coastal areas, including estuaries [15, 16, 23]. The leading economies are agriculture, horticulture, fisheries, animal husbandry, mining, industry, and tourism.

The landscape harbours endangered Asiatic lions (*Panthera leo persica*) and other carnivores such as the Indian leopard (*Panthera pardus fusca*), striped hyena (*Hyaena hyaena*), Indian golden jackal (*Canis aureus*), jungle cat (*Felis chaus*), Indian fox (*Vulpes benghalensis*), honey badger (*Mellivora capensis*), rusty-spotted cat (*Prionailurus rubiginosus*), ruddy mongoose (*Herpestes smithii*), Indian grey mongoose (*Herpestes edwardsii*), small Indian civet (*Viverricula indica*), etc. Major herbivores include sambar, spotted deer, blue bull, hanuman langur, Indian gazelle, and four-horned antelope [24]. Besides the Asiatic lion’s source population of Gir PA and surrounding areas, the landscape has seven satellite populations (1. Mitiyala Wildlife Sanctuary, 2. Girnar Wildlife Sanctuary, 3. South-western coast, 4. South-eastern coast, 5. Savarkundla-Liliya and its adjoining areas of Amreli, 6. Bhavnagar Mainland, 7. Bhavnagar Coast), some of which also became the source populations.

### Methodology

#### Sampling strategy and data collection

Saurashtra landscape consists of a mosaic of protected areas and mixed-used landscapes with different habitat compositions. Therefore, we divided the landscape into eight different study sites to cover the whole landscape across their protection status (PAs vs. non-PAs) and habitat gradient (Table 1). Study sites include four protected areas having woodland habitat, i.e., a) Gir National Park & Wildlife Sanctuary (hereafter Gir) (Fig A in S1 File), b) Mitiyala Wildlife Sanctuary (hereafter Mitiyala) (Fig B in S1 File), c) Paniya Wildlife Sanctuary (hereafter Paniya) (Fig C in S1 File), d) Girnar Wildlife Sanctuary (hereafter Girnar) (Fig D in S1 File), and reserved and unreserved areas having grassland habitat falling under different forest administrative divisions, i.e., e) Grasslands (*vidis*) of Gir (hereafter Gir grasslands) (Fig E in S1 File), f) Grasslands of Junagadh Forest Division (hereafter Junagadh grasslands) (Fig F in S1 File), g) Grasslands of Bhavnagar Forest Division (hereafter Bhavnagar grasslands) (Fig G in S1 File), and coastal forests having dense *Prosopis juliflora* vegetation, i.e., h) Coastal forests (Fig H in S1 File). Later stated division helps compare the population estimates along different protection and habitat gradients (Table 1). Reserved and unreserved areas with grassland habitats are mosaics of patches of different sizes. Therefore, of these, we have considered patches having areas larger than 10 hectares in the study sites, and data obtained from these sites were pooled for further analysis (Tables 6–8 in S2 File).

**Table 1:** Density estimates of wild prey species in different study sites consist of different protection status and habitats in the Saurashtra landscape.

Species	Density (individuals ± SE/km^2^)							
	Protected areas having woodland habitat				Reserved and unreserved areas having grassland habitat			Reserve forests having dense *Prosopis* vegetation
	Gir	Mitiyala	Paniya	Girnar	Gir grasslands	Junagadh grasslands	Bhavnagar grasslands	Coastal forests
**Spotted deer**	58.37±7.05	76.31±8.19	39.02±10.51	31.18±10.80	46.94±11.40	9.45±5.50	8.86±1.42	9.59±3.29
**Sambar**	3.99±0.71	0.16*	0.15*	11.54±1.95	0.15*	NA	0.28*	NA
**Blue bull**	0.65±0.18	2.40±0.28	0.20*	1.12±0.49	9.66±2.73	16.58±5.60	22.39±4.12	21.52±2.59
**Indian gazelle**	0.08±0.04	NA	0.30*	NA	0.65±0.20	0.35*	2.39±0.79	NA
**Four-horned antelope**	0.009*	NA	NA	0.01*	0.02*	NA	NA	NA
**Blackbuck**	NA	NA	NA	NA	0.24*	12.57±6.90	4.87*	NA
**Wild pig**	2.21±0.61	0.60*	0.80*	0.36*	4.33±1.14	7.38±3.89	2.66±1.41	7.13±1.48
**Hanuman langur**	41.62±8.87	NA	1.46*	46.00±27.00	3.27±1.32	NA	NA	NA
**Indian peafowl**	37.01±5.05	40.58±5.23	36.42±10.12	68.87±25.39	18.55±2.48	10.54±3.61	24.12±7.90	11.85±1.84

*Crude density: Density estimates derived from dividing the number of individuals encountered by study area; NA = density not assessed due to low sample size; SE = Standard Error

Road transects have been widely utilized for large-scale surveys because they provide rapid and extensive coverage of the study area [25, 26]. Since our study was carried out at the landscape level, therefore we used road transects to achieve our objectives. However, road transect violates one of the important assumptions of distance sampling, i.e., independent distribution of animals along the line. Nevertheless, it has evolved as one of the most robust scientific methodologies to assess species abundance at the landscape level [26–28]. In addition, it is worth mentioning that certain areas within the study sites lacked a well-developed road network. Therefore, foot transects were walked in such areas to ensure better spatial coverage of the sampling unit for data collection.

Sampling was conducted from 8 May to 20 May 2022 in different study sites (Table 1 in S2 File). The exercise was carried out by regularly employed forest personnel with at least five years of working experience in the landscape. Before data collection, all the forest personnel were trained to familiarize themselves with the methodology, work distributions in the team, observations to be recorded, data sheets, hands-on instruments handling in the field, timings, and transects. To ensure sufficient and sizeable data collection, a total of four temporal replicates of each transect were run. During the transect monitoring, data collected includes the name of species encountered, their group size, age and sex structure of the group, the perpendicular distance from the transect (using Hawke Laser Range Finder 900M), start and end time of the transect, GPS location (recorded using Garmin e-Trex 30), transect length and milometer reading during the encounter. Open four-wheel drive vehicles were used to monitor the road transects. Each vehicle accommodated three observers who divided their data collection responsibilities. Two observers were assigned to collect data on both sides of the transect, while the third observer noted the data. This division of work ensured comprehensive coverage and efficient data collection during the road transect monitoring. Transects were monitored by vehicles running at <20 km per hour to ascertain all the detections on and around the roads. Two observers walked foot transects to collect the data as defined earlier. Transects were monitored during the morning (0600–0900 hours) and evening hours (1600–1900 hours).

Age and sex composition were assessed by categorizing encountered groups (S3 File) into three classes–adult male, adult female and fawn. Adult males and females were distinguished based on their darker coat colour and body size, along with large antlers for males in some ungulate species. Fawns (0–6 months) were easily identified by their very small body size.

#### Data analysis

**Fig 2:**
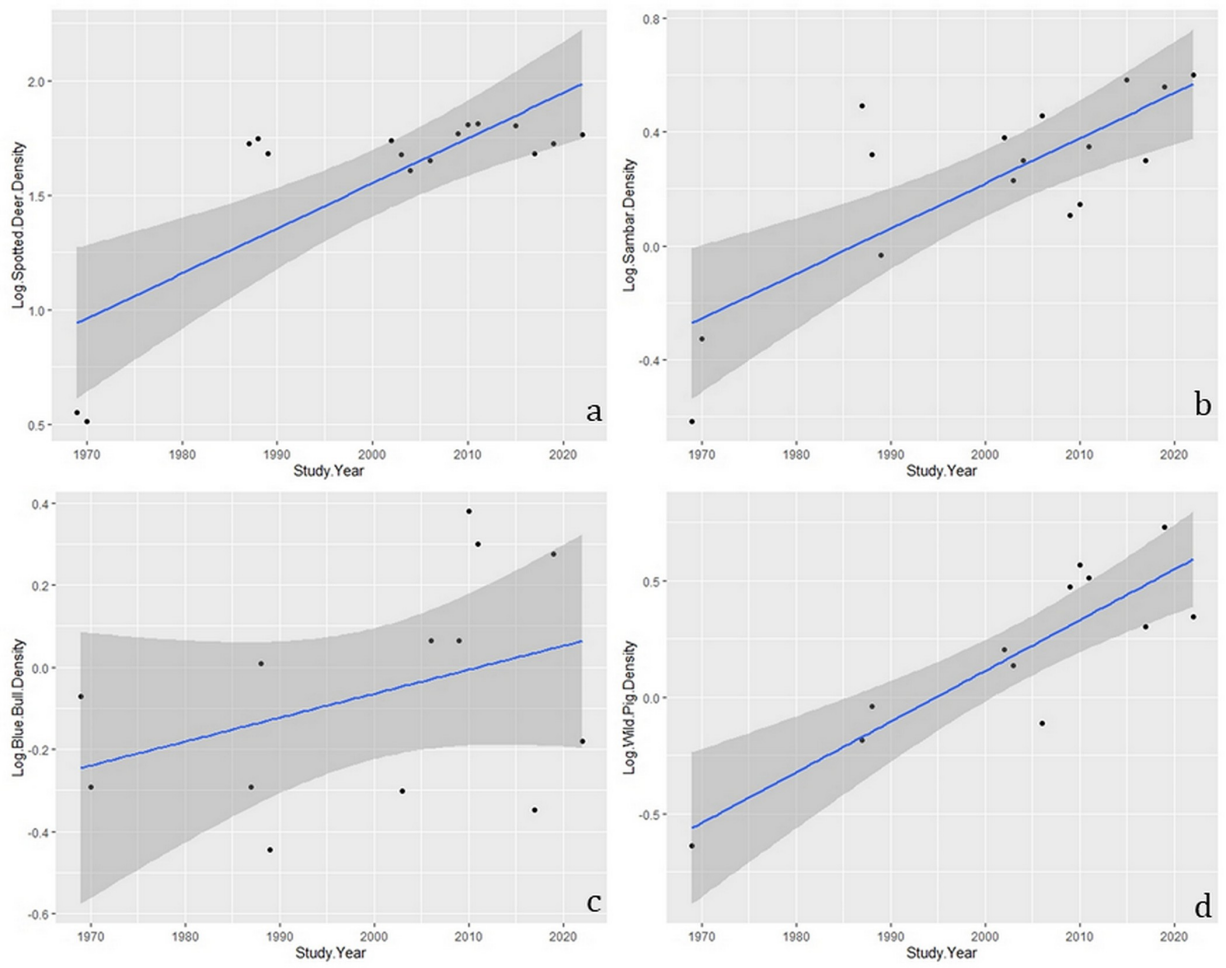
The realized growth rate of spotted deer (a), sambar (b), blue bull (c) and wild pig (d) in Gir National Park and Wildlife Sanctuary.

Data analysis was carried out following the distance sampling framework using the program DISTANCE 7.4 [29, 30]. We used the coefficient of variance as a determinant of sample size adequacy since it shows the variability in data [29]. Data were analyzed first by grouping perpendicular distances in a very small group to detect evasive movement and heaping from the transect [30]. Further, to overcome these biases, perpendicular distances were grouped in broad distance classes to meet the analytical assumption of distance sampling, such as the shoulder. The grouping of data was checked using chi-square statistics [30], and no significant difference was observed in the data. About 5% of the observations lay at a high distance, and acting as outliers were removed by truncation of the data set [31]. A combination of different key functions with adjustment terms was run to assess wild prey species’ densities. Crude density was calculated for species with insufficient encounters by dividing the number of individuals sighted in each study site by the area of the study site. The key function involved Half Normal (HN), Hazard Rate (HR), and Uniform (UN), while the adjustment term includes Cosine (COS), Simple Polynomial (SP), and Hermite Polynomial (HP) (Figs 1–36 in S4 File). The model with the lowest AIC (Akaike Information Criterion) was considered the best [30, 31]. A larger group sighted at large distances can affect the effective strip width (ESW) and, consequently, the density estimates. Therefore, regression analysis, using an inbuilt function in DISTANCE 7.4, was employed to assess the effect of large group size on detection probability. Data collected were pooled at the study site level for each species and analyzed accordingly. Pooling the data at the study site level helped overcome the detection probability issues arising from spatial or temporal (Morning or evening) replicates of transects and foot or vehicle transects. Species biomass was calculated by multiplying wild prey species’ mean adult weight (kg) by their abundance in different study sites (spotted deer 45; sambar 160; blue bull 180; wild pig 45; Indian gazelle 20; four-horned antelope 14; hanuman langur 12; Indian peafowl 5) (weight units were adopted from [25, 26]).

Additionally, the sex ratio was calculated as the number of adult males per 100 adult females (hereafter AM: AF) [32]. This ratio provides insights into the population dynamics and demographics of the studied ungulate species. Furthermore, the ratio of fawns to 100 adult females (hereafter F: AF) was also assessed as an indicator of productivity in ungulates. The AM: AF and F: AF ratios were calculated for each species at each study site, allowing for a comprehensive understanding of the sex composition and reproductive success within the populations.

To assess the realized growth rate (r ± SE) of the wild ungulate prey population in Gir, a regression analysis was conducted by regressing the natural logarithm-transformed density estimates against time. As density estimates from earlier studies in Gir were not available at regular time intervals, density estimates from studies conducted in different years were utilized (S5 File) [33].

## Results

### Sampling effort

A total of 119 vehicle and foot transects were laid in the study area, with a total length of 1181.35 km. These transects were run for four times, making it a total sampling effort of 4725.40 km. (Tables 2–9 in S2 File).

### Population density and biomass

All the protected areas with woodland habitats exhibited high density and biomass of deer species, i.e., spotted deer and sambar, compared to the grasslands and coastal forests. Spotted deer density (individuals±SE/km^2^) was highest in Mitiyala (76.31±8.19) while lowest in Junagadh grasslands (9.45±5.50) (Table 1), whereas the sambar density was highest in Girnar wildlife sanctuary (11.54±1.95) while lowest in coastal forests and Junagadh grasslands (0).

On the contrary, antelopes (blue bull, Indian gazelle and blackbuck) exhibited high density and biomass in the grasslands and coastal forests compared to woodland. Among the study sites, the highest density of blue bull was recorded in Bhavnagar grasslands (22.39±4.12), while the lowest was in Paniya (0.20—crude density). For Indian gazelle, the highest density was found in Bhavnagar grasslands (2.39±0.79), while the lowest was in Mitiyala (0). Junagadh grasslands had the highest density of blackbuck (12.57±6.90), while no sightings of blackbuck were there in the woodland habitat, i.e., PAs.

Wild pig density and biomass were also higher in grasslands than in the protected areas. The highest density of wild pig was in Junagadh grasslands (7.38±3.89), while the lowest was in Mitiyala (0.60—crude density). Due to insufficient sample size, the density of the four-horned antelope could not be assessed in the protected areas, grasslands, and coastal forests.

Hanuman langur showed higher density and biomass in PAs than in the grasslands and coastal forests. Among the study sites, Girnar Wildlife Sanctuary recorded the highest density of hanuman langur (46.00±27.00), while the lowest densities were observed in Junagadh grasslands, Bhavnagar grasslands and coastal forests. In contrast to other species, Indian peafowl was found to be widespread across all the study sites. The highest density of Indian peafowl was observed in Girnar Wildlife Sanctuary (68.87±25.39), while the lowest was recorded in Junagadh grasslands (10.54±3.61) (For further detailed results, see S6 File).

### Growth rate of ungulates in Gir

The realized growth rate for spotted deer in Gir was (0.01±0.004, p<0.05; r^2^ = 0.60) (Fig 2A) while for sambar was (0.01±0.003 p<0.05; r^2^ = 0.60) (Fig 2B). In the case of blue bull, the realized growth rate was (0.005±0.004, p>0.05; r^2^ = 0.14) (Fig 2C), and for wild pig was (0.02±0.003, p<0.05; r^2^ = 0.75) (Fig 2D).

### Age and sex ratio

The AM: AF ratio was found to be female-biased for all the ungulate species at all study sites except for blue bull and Indian gazelle in Gir, where the sex ratio was male-biased (Table 2). Hanuman langur also showed a female-biased sex ratio in all the study sites. On the other hand, Indian peafowl exhibited a male-biased sex ratio in the grasslands and coastal forests, while it was female-biased in woodlands. Spotted deer and wild pig showed higher F: AF ratios compared to sambar, blue bull, and Indian gazelle (Table 3). However, due to the very low sample size, AM: AF and F: AF ratios were not calculated for blackbuck and four-horned antelope. Hanuman langur also exhibited a high F: AF ratio.

**Table 2:** Adult male to 100 adult female ratio at different study sites.

Species	AM: 100 AF ratio at different study sites							
	Gir	Mitiyala	Paniya	Girnar	Gir grasslands	Junagadh grasslands	Bhavnagar grasslands	Coastal forests
Spotted deer	39	38	63	59	32	29	39	36
Sambar	44	NA	NA	48	NA	NA	NA	NA
Blue bull	136	45	NA	53	50	37	43	45
Indian gazelle	105	NA	NA	NA	75	NA	66	NA
Four-horned antelope	NA	NA	NA	NA	NA	NA	NA	NA
Blackbuck	NA	NA	NA	NA	NA	38	NA	NA
Wild pig	63	NA	NA	NA	83	57	70	74
Hanuman langur	43	NA	NA	38	40	NA	NA	NA
Indian peafowl	82	70	47	67	101	117	121	113

*NA = Not Assessed due to low sample size.

**Table 3:** Fawn to 100 adult female ratio at different study sites.

Species	F: 100 AF ratio at different study sites							
	Gir	Mitiyala	Paniya	Girnar	Gir grasslands	Junagadh grasslands	Bhavnagar grasslands	Coastal forests
Spotted deer	18	8	26	13	17	17	32	11
Sambar	20	NA	NA	11	NA	NA	NA	NA
Blue bull	20	NA	NA	3	6	4	10	14
Indian gazelle	5	NA	NA	NA	10	NA	7	NA
Four-horned antelope	NA	NA	NA	NA	NA	NA	NA	NA
Blackbuck	NA	NA	NA	NA	NA	16	NA	NA
Wild pig	60	NA	NA	NA	45	32	174	89
Hanuman langur	77	NA	NA	45	40	NA	NA	NA
Indian peafowl	NA	NA	NA	NA	NA	NA	NA	NA

*NA = Not Assessed due to low sample size.

## Discussion

### Population density and biomass

Our hypothesis was partially supported as not all prey species exhibited high densities in PAs. Deer species (spotted deer and sambar) and hanuman langur showed high density in the woodland habitat of PAs, while antelopes showed high density in the grasslands. Indian peafowl density was higher in the PAs compared to the grasslands and coastal forests.

Factors that affect population density and abundance of wild prey ungulates include forest vegetation types, topography, diet, forage availability, body size, sociality, human disturbances and predation by large carnivores [25, 27, 34–38]. Spotted deer and sambar showed comparatively higher density in the woodland ecosystem of protected areas compared to grasslands. Our findings corroborate with the findings for the same species from the semi-arid ecosystem of Ranthambore National Park in India [39]. Several scientific studies have found spotted deer prefer flat terrain, highly nutritious forage availability, and early succession woodland vegetation with open cover [25, 33, 37, 40, 41], while sambar have a strong preference for hilly terrain and dense habitat [25, 27, 38, 39, 40, 42, 43]. Gir, Paniya and Mitiyala have availability of highly nutritious forage in the form of *Ziziphus*, *Carris*, *Capparis* and *Acacia* species, and the western and eastern parts of Gir have relatively flatter terrain [25], which helps spotted deer to achieve high density. Also, being highly gregarious and having a tendency to form large-size groups may help spotted deer attain high density. On the other hand, among the PAs, Girnar has high hilly terrain along with dense habitat, while the latter stated habitat in Gir is limited to the central part, and the western and eastern regions have relatively flatter terrain and moderate open habitat. Hence, the high availability of preferred habitats in Girnar might lead to the high density of sambar in Girnar compared to Gir.

However, spotted deer densities were comparatively low in grasslands and coastal forests. Grasslands situated in multi-use landscape matrix have historically been exploited for fodder, agriculture practices, and grazing, leading to fragmentation and a decline in grassland quality [16]. Also, hunting in these grasslands during the pre-historic era was among the favorite sports of royal families [16]. Grassland restoration in Saurashtra is a recent phenomenon (around two decades ago). It will take time for the grasslands to reach their full ecological potential in terms of quantity and quality. Also, most of the grasslands are scattered in small patches rather than continuous patches of habitat and hence have limitations in sustaining the high density of spotted deer. As a management strategy and goodwill gesture (supplied to drought-affected areas and local people for supporting wildlife conservation) activity, the management authorities harvest the grasses in the early winter season (end of October to mid-January), especially in reserved grasslands. This practice of grass harvesting may enhance the quality of the grasses by promoting the growth of new sprouts [44], as the nutritive value of the grass depends on its maturity stage. Therefore, the management authorities’ practice of grass harvesting may improve the palatability and availability of grasses for herbivores.

On the other hand, coastal forests have dense patches of mainly *Prosopis juliflora*, which offer inadequate nutrition for population growth and do not fit in the open habitat requirements of spotted deer and hence have low densities. The simple explanation for the very low density of sambar in grasslands and coastal forests is the lack of dense habitat with hilly terrain.

Antelopes worldwide are strongly associated with grasslands and savanna habitats, as their recent evolutionary radiation closely corresponds to the spread of savanna habitats globally [45–47]. The findings of our study also support the above findings. All the studied antelope species (blue bull, Indian gazelle, four-horned antelope, blackbuck) showed higher density in grasslands than in woodlands. Among studied antelopes, only blue bull achieved higher density than other species. The formation of large groups is an important factor contributing to the high density of blue bulls in grasslands compared to woodland ecosystems. Habitat openness and forage availability are among the crucial variables that affect antelope group sizes [48, 49]. Grasslands, characterized by their open habitat and high visibility, enable strong coordination among individuals and facilitate large-group formation. In contrast, woodlands, being the dense habitat, offer low visibility and limit coordination between individuals, resulting in comparatively small group sizes [50]. Due to their large size, blue bulls have lower metabolic rates and can sustain themselves on a coarse diet consisting of grasses, which are abundant in grassland habitats. Therefore, their generalist diet, coupled with high forage availability and preference for open habitats like grasslands, allows blue bulls to thrive at higher densities in open habitats compared to woodlands like Gir and Girnar. Also, PAs have a very high density of leopards and lions, and blue bulls contribute highly to the diet of both predators; therefore, predation by leopards and lions might be another key reason for their low density in PAs [51, 52].

Blackbuck is another medium-sized antelope and a group living grazer in the Indian subcontinent [50, 53]. Its exclusive presence in the grassland ecosystem within the study area suggests specialized resource requirements for food. However, only Junagadh grasslands have favorable blackbuck density among the grasslands sampled. The rest of the grasslands have low density, and the sample size was insufficient to estimate density. As stated earlier, for spotted deer, the blackbuck population has faced historical disturbances like poaching, sports hunting and other anthropogenic pressure. It will require time for the blackbuck population to recover and potentially reach higher densities.

Indian gazelle and four-horned antelope are two small antelopes that exist at very low densities in the study area. We did not even get enough data for density estimation of four-horned antelope across the study sites, possibly due to their highly elusive behaviour. Being small in size, both the Indian gazelle and four-horned antelope have high metabolic rates, which require a high-quality diet [48]. A high-quality diet is not easily available and might constrain these species’ population growth compared to coarse feeders like the blue bull and spotted deer [48]. Therefore, due to factors like habitat degradation, grasslands have a meagre availability of high-quality browsable species, resulting in the low population density of the Indian gazelle [39, 54, 55]. Moreover, the historical disturbances mentioned earlier for blackbuck and spotted deer also apply to the Indian gazelle since this species historically shared the same landscape. Four-horned antelope, apart from a dependency on a high-quality diet, is territorial and forms a pair bond that rules out the chance of a high congregation of individuals like spotted deer [56–59]. All these latter-stated factors might contribute to our study area’s rare status of the four-horned antelope.

Wild pig is another ungulate species under study and showed higher density in grasslands than woodland. It is a prolific breeder and diet generalist and can exist in various habitats. In our study area, grasslands are embedded in the agro-pastoral matrix, and it is quite possible that wild pigs can easily exploit agriculture fields for key resources such as food and may use grasslands for resting/breeding purposes. Therefore, the easy gathering of resources might increase wild pigs’ population density in the grasslands.

The hanuman langur exhibits a higher density in woodlands compared to grasslands. This can be attributed to the arboreal nature of the langur, as the limited availability of trees in grassland contributes to the low density of hanuman langurs in such areas. In contrast, woodlands provide a more suitable environment for the langurs due to the presence of trees, allowing them to thrive and maintain a higher population density.

The density of Indian peafowl was found to be higher in woodlands compared to grasslands. This can be attributed to the fact that Indian peafowl, being a large bird, is vulnerable to predation by various predators [26]. Woodlands, with their dense understory, offer a secure cover for resting during the day, providing protection against potential predators. The tree canopy in woodlands also provides secure roosting sites at night, which may not be readily available in grassland habitats. The presence of suitable cover in woodlands enhances the overall survival of Indian peafowl, leading to a higher population density in these woodland habitats compared to grasslands.

### Age and sex ratio

The adult males to 100 adult females ratio among all the ungulate prey species was found to be female-biased except for blue bull and Indian gazelle in Gir, where it was male-biased. Female biased sex ratio in the case of all the ungulate prey species under study has been supported by some earlier studies, too [60–63]. The male-biased sex ratio of blue bull and Indian gazelle was reported by Bagchi et al. (2008) [32] from the Ranthambore tiger reserve. The skewed sex ratio towards females in ungulates might be a result of disproportionate high mortality among males due to factors like intraspecific competition for mates and their selective predation. The male-biased sex ratio among ungulates arises due to sex differences in dispersal or immigration propensity [64] or in the survival of individuals between birth and adulthood due to sex differences in processes such as dispersal, territory acquisition or reproduction [65]. Regan et al. (2020) [66] recently found a male-biased sex ratio in the feral horse population due to predominantly male-biased adult survival. We believe that further research is needed in the case of the blue bull and Indian gazelle in Gir to understand the process of the male-biased sex ratio fully.

Spotted deer, sambar and wild pig showed a higher fawn to 100 female ratio than blue bull and Indian gazelle. A similar high ratio of sambar and spotted deer was also reported by Ramesh et al. (2011) [61] from the Mudumalai tiger reserve, India. The high fawn-to-female ratio in the case of spotted deer, sambar and wild pig indicates their higher productivity than blue bull and Indian gazelle. However, our sampling period was less than one month, which might not coincide with the birthing peak of all the ungulates and hence, the results might not represent the actual difference in the fawn to 100 female ratio.

### Ungulate population growth in Gir

In Gir, spotted deer and sambar showed minimal but positive growth rates, indicating that the population of both species have reached the state of stabilization with very little change. Minimal variation in the spotted deer population from the last three decades indicates stabilization of its population at a high number. Such high density and slight variation indicate that the Gir is sustaining the high spotted deer population (Fig 2A). An earlier study [67] concluded that such a high increase in spotted deer density is due to a decrease in competition with livestock by the resettlement of *maldhari nesses* (temporary human settlement of pastoralist community) outside the Gir, which led to an increase in forage availability. However, population theory states that high population density can lead to intraspecific competition for forage, potentially resulting in a population decline or negative growth rate [68]. Nevertheless, the consistency of the population at high density for more than three decades indicates excellent forage availability; otherwise, one could expect a decline in the spotted deer population, as mentioned above. Such high forage availability could result from increased rainfall in Gir during the last three decades (S7 File).

On the contrary, sambar showed a minimal positive growth rate, and its population showed more fluctuation than spotted deer since the population estimates of 1987 [67]. The highest density level that sambar has achieved over three decades is 3–4 individuals km^-2^. Such low-density equilibrium of sambar for more than three decades is possibly due to high predation by large predators like Asiatic lions (prefer large-size prey) and Indian leopards (a chance hunter and prefers small to medium size prey which the sambar fawn and sub-adults are) and also specific habitat preference. Spotted deer and sambar are mixed feeders in Gir [69]. If we consider sambar density is limited by forage availability, it should have also limited the spotted deer population at low density. Therefore, we negate the possibility of forage availability limiting the sambar density in Gir. Blue bull showed high fluctuations in population with no significant overall change over the last four decades in Gir. Regression graphs indicate that the populations of both ungulates attempt to increase but then sharply decline. Such high fluctuations indicate that some limiting factors are acting and need further research. Wild pigs showed a positive growth rate with an increasing population. Wild pigs during the 1970s and 80s had a low population. An increase in the population of wild pigs might indicate its release from the limiting factors that affected them in the past. However, further research is needed to assess the specific limiting factors influencing the wild pig population in Gir.

## Conclusion

In conclusion, our study revealed that the density and abundance of wild prey species are influenced by a combination of factors, including their evolutionary affinity with the habitat, distribution of habitats in the study area, protection status, and grouping tendencies. While no single biological or physical factor was found to directly affect the pattern of density and biomass of wild prey species, it is the interaction and combination of these factors that shape the observed patterns. Therefore, a holistic understanding of these factors is important for comprehending the dynamics of wild prey populations.

## Conservation implications

The present study has substantial conservation implications for ungulates at the landscape level. Protection and disturbance in different study sites are among the key factors responsible for the density gradient of ungulates apart from their habitat preferences and social organization. Therefore, ensuring effective protection measures in non-protected areas is crucial to minimize human disturbances. Effective protection would help in increasing the quality of the grasslands and coastal forests surrounding PAs and would benefit the prey population positively. The observation of male-biased sex ratios in species such as blue bull and Indian gazelle in Gir raises concerns regarding future population dynamics. Therefore, it is essential to investigate the factors influencing the sex and age ratios of prey species. In Gir, ungulate populations exhibited minimal growth rates, with spotted deer maintaining high densities while sambar, blue bull, and wild pig at low densities. There is a need to assess the ecological factors limiting the population of wild ungulates in Gir since the later stated ungulate species are among the important prey for lions and leopards in Gir, acting as a source population for the surrounding landscape. The small sample size obtained for some ungulate species, such as the four-horned antelope, poses challenges to their proper conservation. Distance sampling techniques may not perform well with small sample sizes, highlighting the need to explore alternative methods in future studies. For example, adopting camera trap-based distance sampling could provide more accurate population estimates for species like the four-horned antelope.

The present study also has key implications for conserving large carnivores (Asiatic lions and Indian leopards) and their habitats in the Asiatic Lion Landscape. Gir acts as the source population for Asiatic lions, and the surrounding landscape acts as a sink habitat for dispersing and growing lion populations. In order to sustain the lion population in the landscape, it is crucial to have accurate estimates of the ungulate population in the landscape, which the present study has provided. The study has also revealed that various factors, including species-specific life history traits such as social organization and group size, dietary specialization, and variations in terrain and habitat availability, influence the gradient of wild ungulate density in the study area. These factors may be considered when formulating management strategies to sustain the ungulate population in the landscape. The findings of this assessment can also aid in prioritizing conservation efforts for areas with high wild ungulate density, particularly in forest patches that act as stepping stones in the landscape. Such areas need special conservation emphasis as they play a crucial role in supporting the large carnivore populations in the landscape.

## Supporting information

S1 File Maps showing transects in different study sites in Asiatic Lion Landscape, Gujarat, India. (DOCX)

S2 File Details of phases, study sites, time duration and transects laid in different study sites in Asiatic Lion Landscape, Gujarat, India. (DOCX)

S3 File Number of groups encountered during the sampling period at different study sites. (DOCX)

S4 File Detection probability and distance data for different wild prey species in different study sites in Asiatic Lion Landscape, Gujarat, India. (DOCX)

S5 File Density estimates used in the calculation of realized growth rate using regression. (DOCX)

S6 File Detailed results of wild prey species at different study sites. (DOCX)

S7 File The increasing trend of average rainfall in Gir National Park and Wildlife Sanctuary. (DOCX)

## Abbreviations

UN: Uniform
HR: Hazard rate
HN: Half Normal
COS: Cosine
D: Density per km^2^
SE: Standard Error
MGS: Mean Group Size
GD: Group Density (per km^2^)
GER: Group Encounter Rate (per km)
CI: Confidence Interval
CV: Coefficient of Variance
ESW: Effective Strip Width
NA: Not assessed due to small sample size
