# Supplementary material for: The semi-arid ecosystem of Asiatic Lion Landscape in Saurashtra, Gujarat: Population density, biomass and conservation of nine wild prey species
Source: PLoS One. 2023 Sep 28;18(9):e0292048. doi: 10.1371/journal.pone.0292048 (PMC10538734; doi:10.1371/journal.pone.0292048)
Supplement: S1 File — (DOCX) [file pone.0292048.s001.docx]

**Maps showing transects in different study sites in Asiatic Lion Landscape, Gujarat, India.**


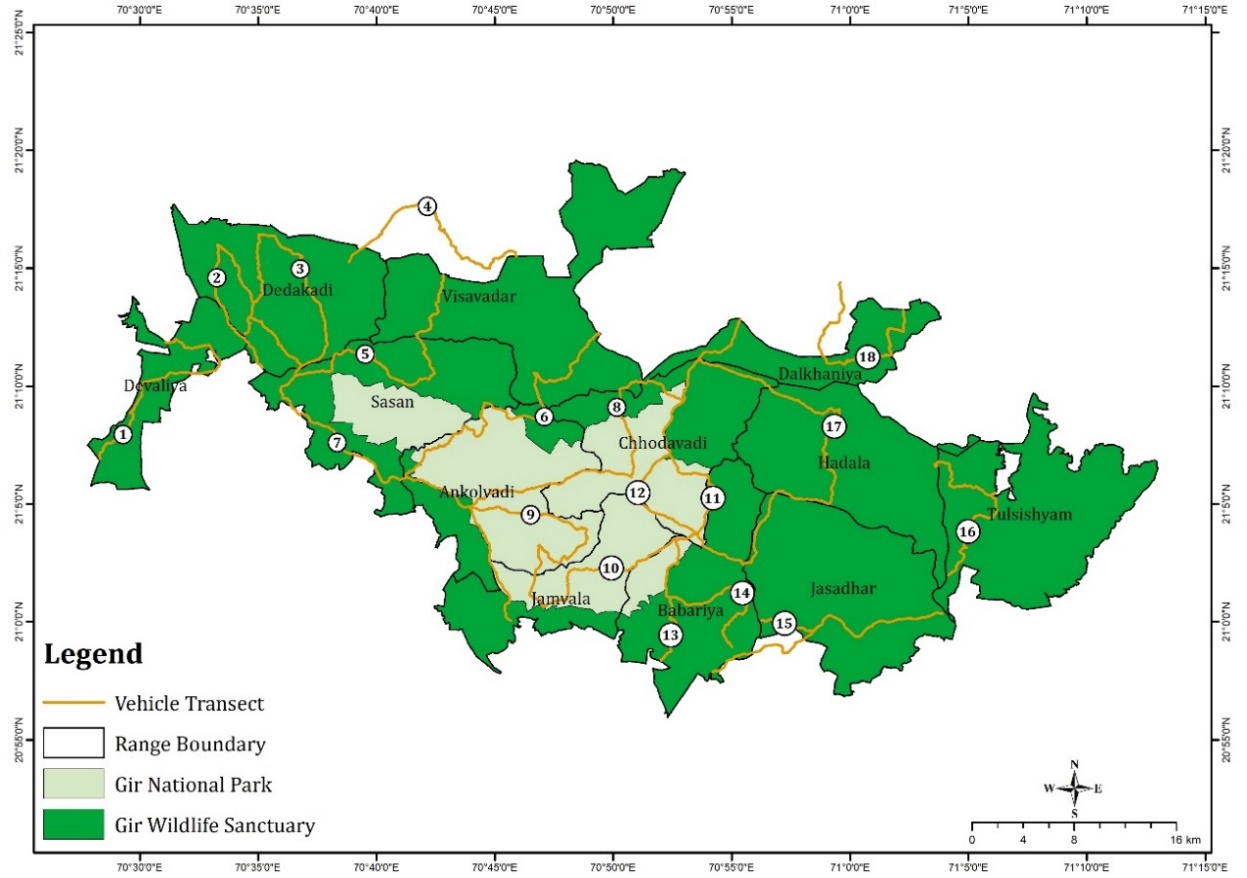


**Fig. A: Map indicating vehicle transects in Gir National Park and Wildlife Sanctuary.**


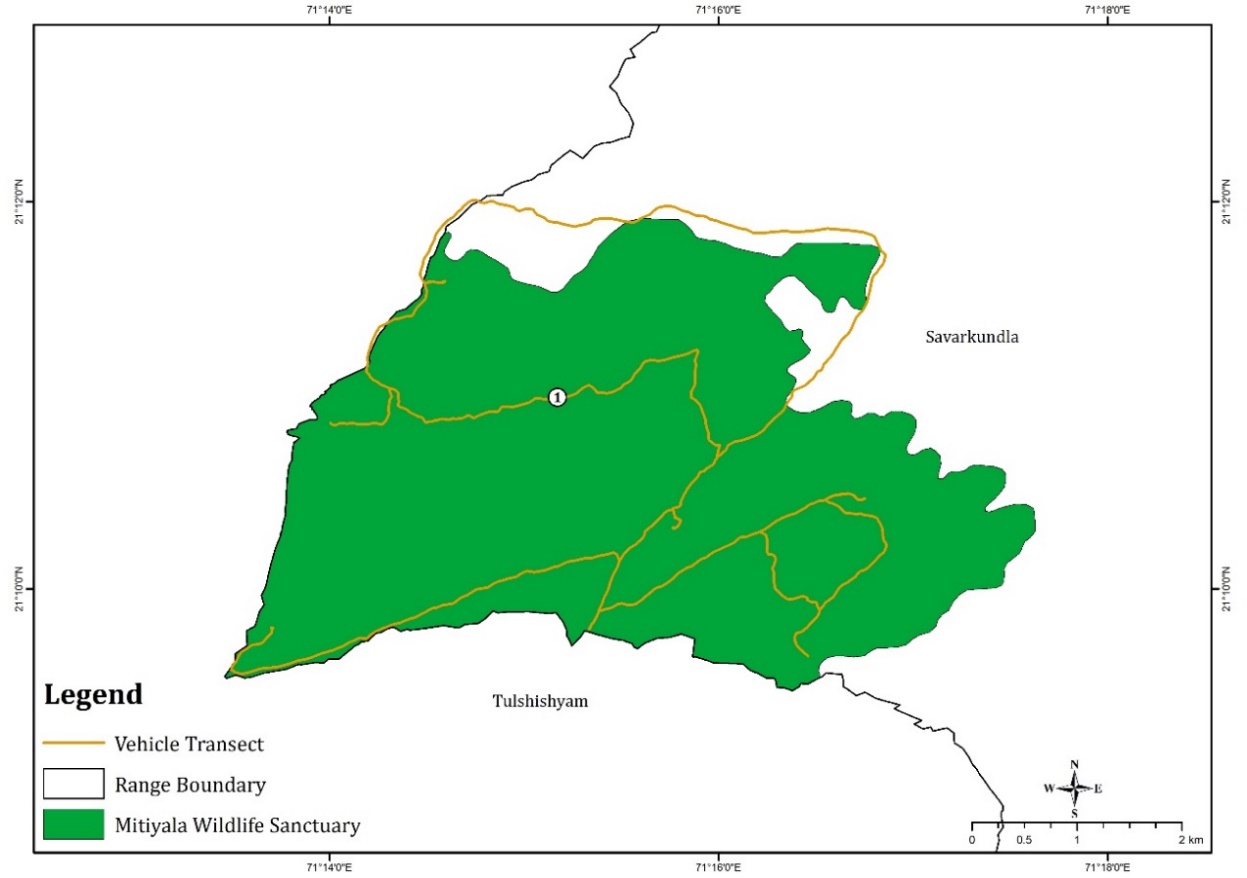


**Fig. B: Map indicating vehicle transects in Mitiyala Wildlife Sanctuary.**


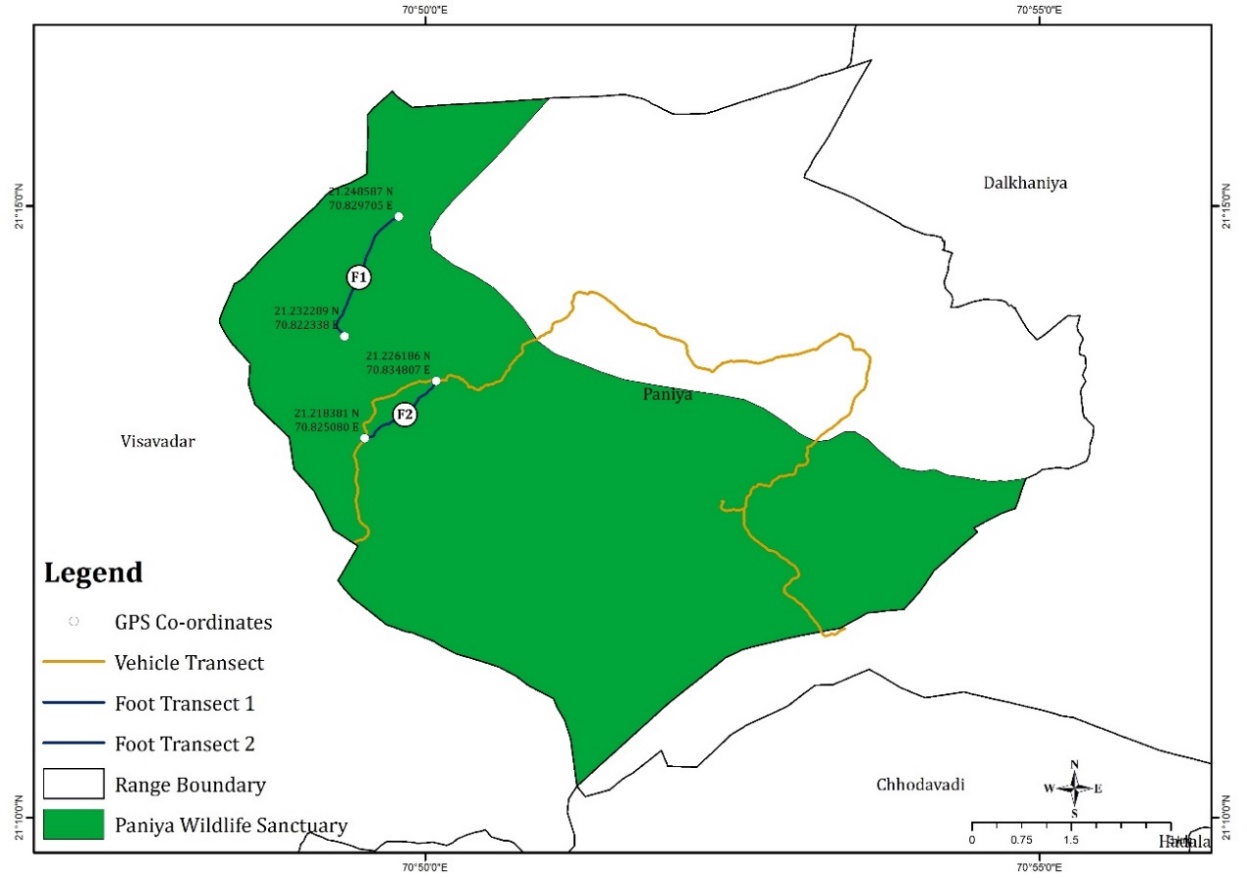


**Fig. C: Map indicating vehicle transects in Paniya Wildlife Sanctuary.**


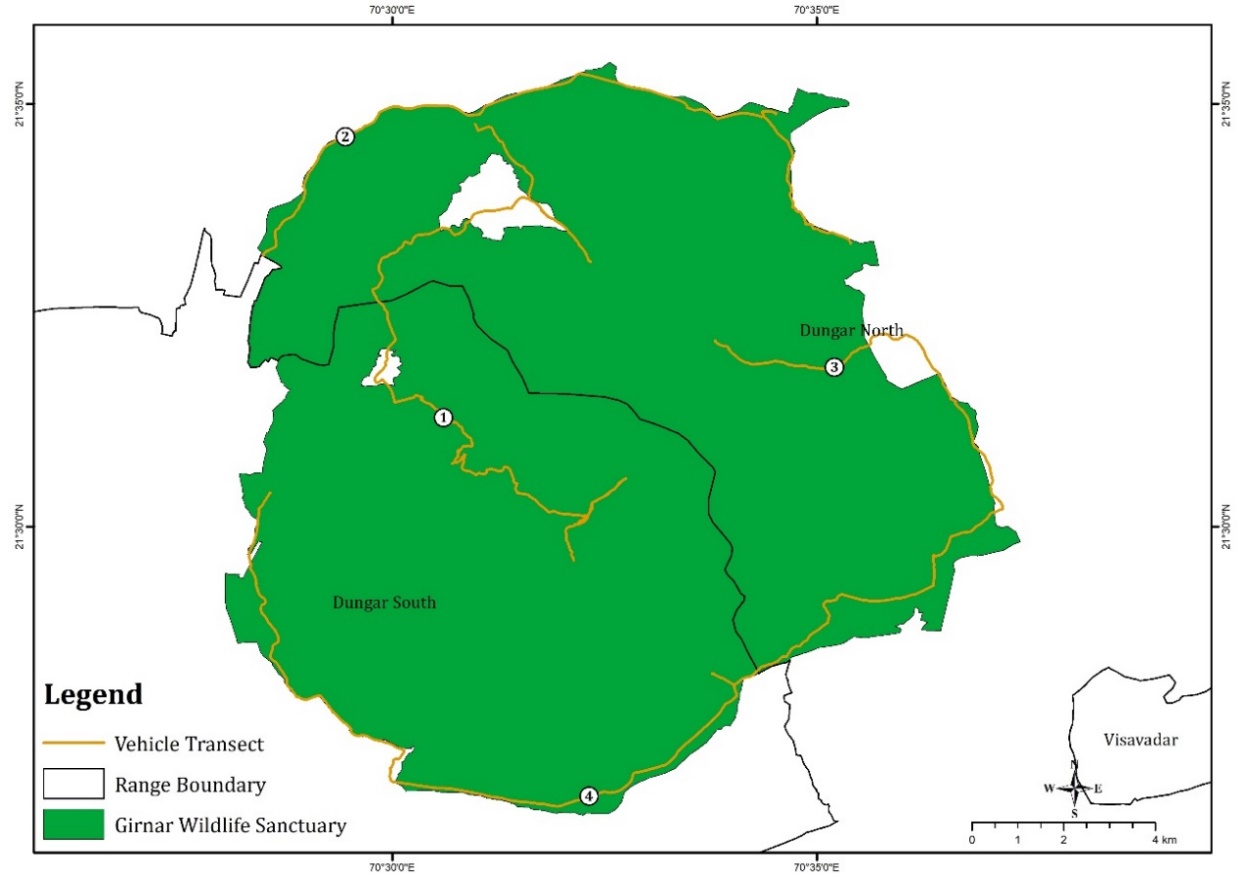


**Fig. D: Map indicating vehicle transects in Girnar Wildlife Sanctuary.**


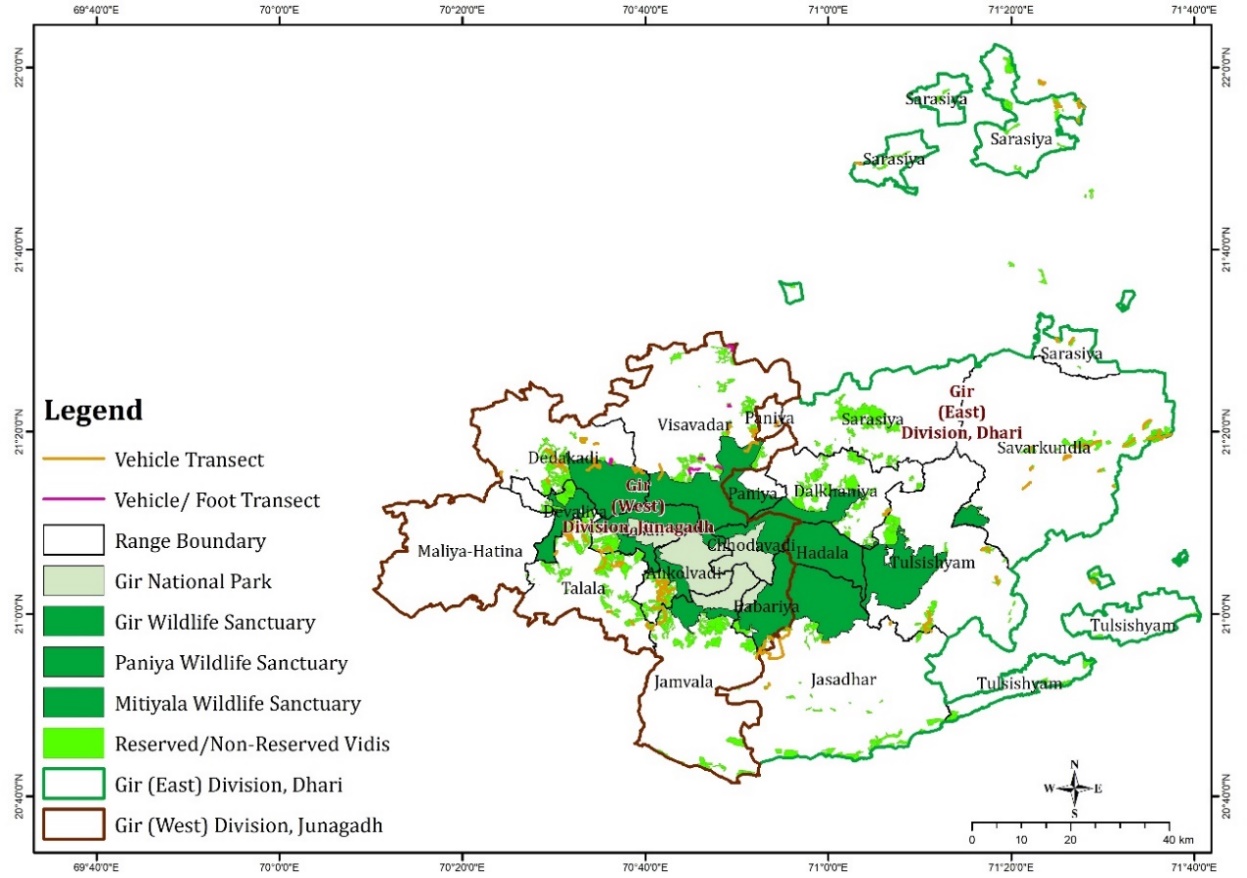


**Fig. E: Map indicating vehicle/foot transects in Gir grasslands.**


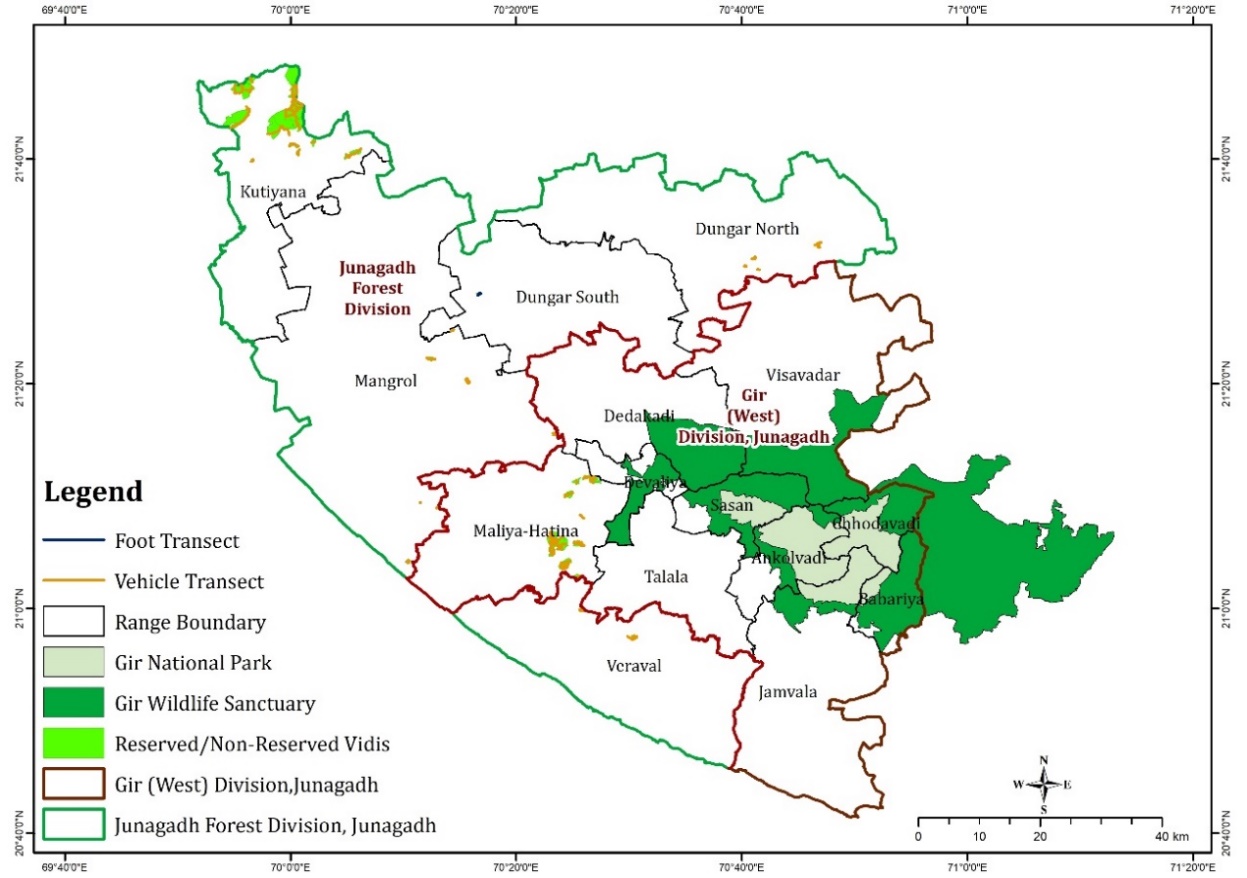


**Fig. F: Map indicating vehicle/foot transects in Junagadh grasslands.**


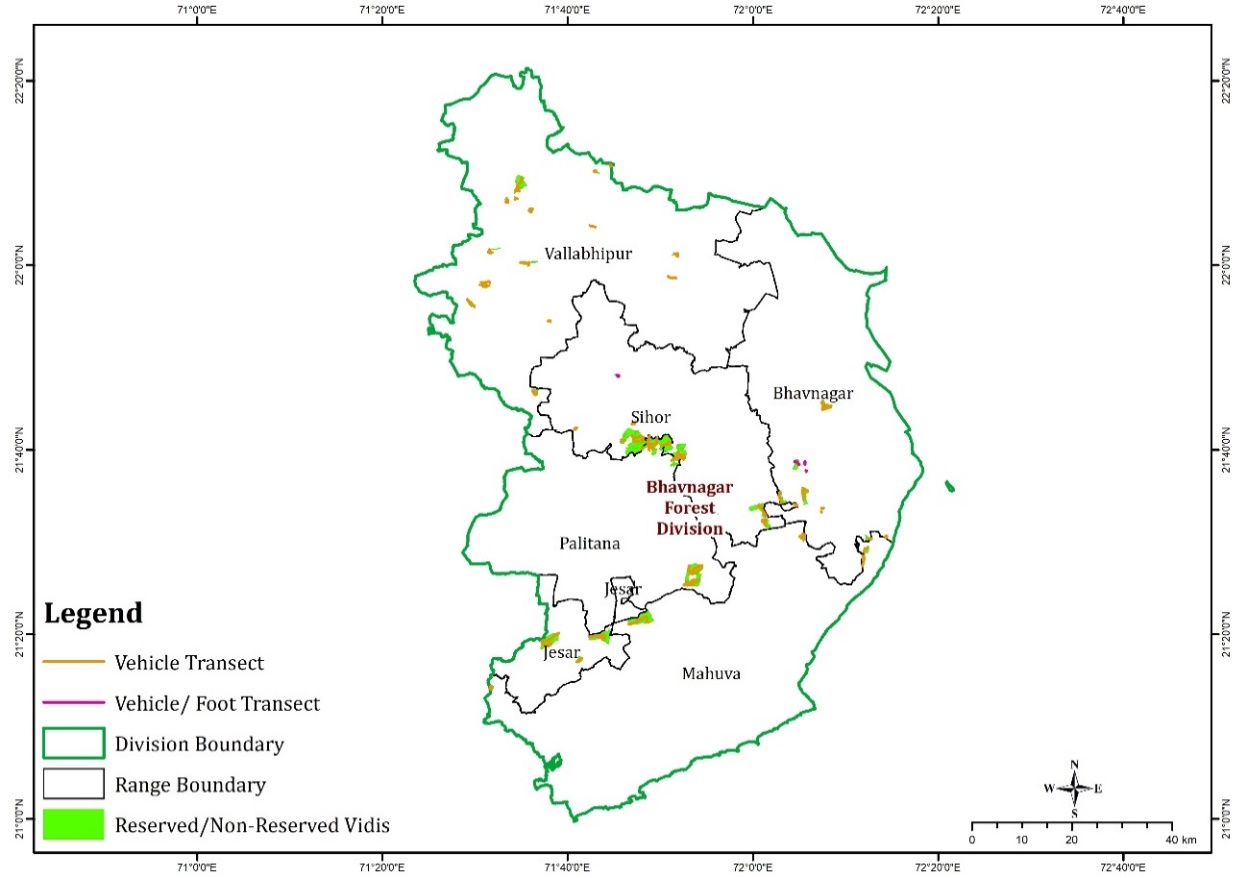


**Fig. G: Map indicating vehicle/foot transects in Bhavnagar grasslands.**


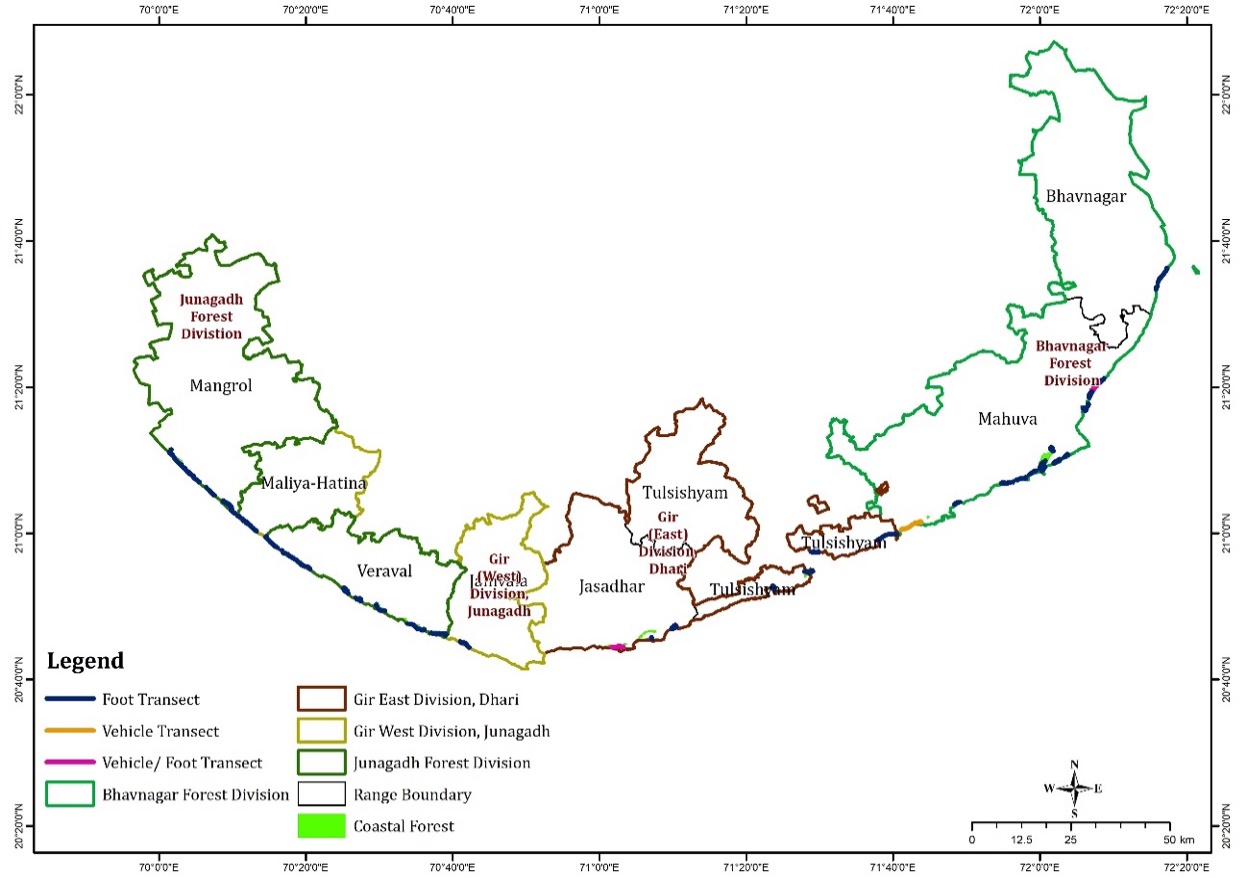


**Fig. H: Map indicating vehicle/foot transects in Coastal forests of Asiatic Lion Landscape.**

*****
