# Supplementary material for: The semi-arid ecosystem of Asiatic Lion Landscape in Saurashtra, Gujarat: Population density, biomass and conservation of nine wild prey species
Source: PLoS One. 2023 Sep 28;18(9):e0292048. doi: 10.1371/journal.pone.0292048 (PMC10538734; doi:10.1371/journal.pone.0292048)
Supplement: S2 File — (DOCX) [file pone.0292048.s002.docx]

**Details of phases, study sites, time duration and transects laid in different study sites in Asiatic Lion Landscape, Gujarat, India.**

**Table 1. Details of phases, study sites, transects and time duration of wild prey population estimation in the Asiatic Lion Landscape in May 2022.**

| **Sr. No.** | **Phase** | **Area** | **No. of**  **transects** | **Transects total length (km)** | **Date of estimation** |
| --- | --- | --- | --- | --- | --- |
| 1. | I | Gir National Park and Wildlife Sanctuary | 18 | 1650 | 08/09 May |
| 2. |  | Paniya Wildlife Sanctuary | 03 | 88 | 08/09 May |
| 3. |  | Mitiyala Wildlife Sanctuary | 01 | 113.60 | 08/09 May |
| 4. | II | Reserved and non-reserved grasslands of Gir (West) and (East) Division | 23 | 990.60 | 10/11 May |
| 5. | III | Girnar Wildlife Sanctuary | 04 | 314.60 | 13/14 May |
| 6. |  | Reserved and non-reserved grasslands of Junagadh Forest Division | 11 | 435.60 | 13/14 May |
| 7. | IV | Reserved and non-reserved grasslands of Bhavnagar Forest Division | 19 | 533.60 | 16/17 May |
| 8. | V | Coastal Forests from Bhavnagar to Mangrol | 40 | 599.60 | 19/20 May |

**Table 2. Details of vehicle transects in Gir National Park and Wildlife Sanctuary.**

| **Transect  No.** | **Transect Details** | **Length (km)** |
| --- | --- | --- |
| 1 | Abudi - Bheriya - Sandhbeda - Gadakiya - Dholivokali - Sakhtimbhi - Charchowk | 19.7 |
| 2 | Sasan - Ratanghuna - Pilipat - Kadeli - Navi Raidi - Paraviya - Kerambha - Khada - Juniraidi - Bhambhafod check post | 27.1 |
| 3 | Khutani - Mandirvali Raidi - Dudhala - Dedakadi - Jambuthala - Sandhiyagali - Dadhiyaness - kankai checkpost | 25.2 |
| 4 | Kansiya - Piyava - Badak - Dudhala - Rampara - (outside) | 19.3 |
| 5 | Sasan - Bavalvala chowk - Valadara - Midholivala - Kamleshwar - Piplavali Aati - Varvangada - Karamdadi - Kutiya | 25.7 |
| 6 | Devadungar - Kissa Vansali - Devkaniya - Kankai mandir - Safra - Lilipani | 29.9 |
| 7 | Sasan - Khokhra - Sirvan - Janvadala | 20.5 |
| 8 | Janvadala - Gola - Bhuvatirth - Bhantha - Rampari - Sapness | 29.6 |
| 9 | Singoda dam - Suki Khodiyar- Vaghatimbi - Kalipat - Vijanand ni Ghidi - Janvadla - Batheshwar - Jamvala | 32.1 |
| 10 | Jamvala - singoda dam - Dabhala- Ambavali Khodiyar - Banej char chowk | 17.2 |
| 11 | Banej char chwok - Lapatani - Vakumbha - Chodavadi no pool | 10.7 |
| 12 | Sapness - chodavadi - Banej char chowk - Machhundari river | 23.5 |
| 13 | Babariya - Patla - Banej char chowk | 13.2 |
| 14 | Popatadi - Ambakhai - Viragali - Patla | 12.8 |
| 15 | Jakhiya - Mandavi - Ghodavadi - Jasadhar | 30.3 |
| 16 | Chikhal kuba - Surnala - Rajasthali - Rebdipat - Loki - Timbarva | 18.6 |
| 17 | Semardi - Sapness - Jamvali - Hadala - Aral - Kardapan machundri river | 37.9 |
| 18 | Sarasiya - Karamdadi - Matanmaal - Govindpur outside | 19.1 |

**Table 3. Details of vehicle transect laid in Mitiyala Wildlife Sanctuary.**

| **Transect  No.** | **Transect Details** | **Length (km)** |
| --- | --- | --- |
| 1 | Mitiyala Wildlife Sanctuary | 28.4 |

**Table 4. Details of vehicle/foot transects laid in Paniya Wildlife Sanctuary.**

| **Transect  No.** | **Transect Details** | **Length (km)** |
| --- | --- | --- |
| 1 | Foot Transect-1 | 2.1 |
| 2 | Foot Transect-2 | 1.4 |
| 3 | Bherai kui-Paniya Sanctuary-Chanchai-Paniya-Lilapani | 18.5 |

**Table 5. Details of vehicle transects laid in Girnar Wildlife Sanctuary.**

| **Transect  No.** | **Transect Details** | **Length (km)** |
| --- | --- | --- |
| 1 | Bordevi-Khodiyar Ghodi-Bhavnath-Intva gate-Intva ghodi-Bhandargala-Jhinabavani madhi-Jambudi | 25.3 |
| 2 | Indreshvar-Jambudi-Paturan-Tanka-Pitaliya Gate | 17.8 |
| 3 | Sarakadiya-Surajkund-Patvad-Kalagadba-Ratanpara-Ransivav-Mathura-Ramnath | 17.4 |
| 4 | Ramnath-Lambdidhar-Dungarpur-Dayanath-Dedakadi-Khodiyar thana-Samudri Gate | 18.1 |

**Table 6. Details of vehicle/foot transects laid and area of Gir grasslands.**

| **Transect  No.** | **Transect Details** | **Area**  **(ha)** | **Length (km)** | **Transect  Type** |
| --- | --- | --- | --- | --- |
| **1** | Isadhar, Ghado | 209.78 | 3 | Vehicle |
| **2** | Roniya, Dhron, Pyava Towers, Chameli Santokadi, Chotakiyali, Vanga Sanga | 1664.05 | 13.7 | Vehicle |
| **3** | Lashkar Shobhavadala, Haripur Towers | 142.15 | 2.5 | Vehicle |
| **4** | Manadiya, Javaldi, Zanjesar pati, Rajpara Towers | 860.38 | 4.18 | Vehicle |
| **5** | Monvel Fulvadi, Sai, Hathaliyo, Lamdhar, Nani Chavandi, Lilya Nadakiya | 353.19 | 11.37 | Vehicle |
| **6** | Moti Chavandi | 518.04 | 9 | Vehicle |
| **7** | Zinzuda, Natadiya, Khodiyar, Malanka | 830.84 | 8.2 | Vehicle |
| **8** | Gadhali, Kendipur, Siyaradi | 240.31 | 4.9 | Foot |
| **9** | Haripur Sajiya, Guda Jaliya | 337.49 | 10.17 | Vehicle |
| **10** | Chitravad, Borvav, Lakkadvera, Sangodra, Lushala, Madhupur, Jasapur, Jepur, Bhojde | 2039.85 | 14 | Vehicle |
| **11** | Rampara, Hadmatiya, Javantri, Mandorana | 1063.72 | 15.4 | Vehicle |
| **12** | Vadala, Bamanasa | 789.15 | 16.74 | Vehicle |
| **13** | Fareda | 225.08 | 5 | Vehicle |
| **14** | Nani Liliya, Boringda, Sariyo, Sandhiyo, Salemar, Vikram | 589.81 | 15.67 | Vehicle |
| **15** | Khambhala, Randal na Dalava | 85.22 | 5.23 | Vehicle |
| **16** | Karangsa, Hirva | 245.92 | 30 | Vehicle |
| **17** | Samadhiyala, Dhrabavad, Bediys, Gir Gadhala, Rasulpara, Chikhli | 354.76 | 10.07 | Vehicle |
| **18** | Makhaniya, Ambaliyala, Dadli | 834.66 | 15 | Vehicle |
| **19** | Sarkdiya, Raidi, Pati, Jhanjharda | 188.75 | 4.3 | Vehicle |
| **20** | Kodiya, Gidardi, Piplava, Khadhar | 722.41 | 15.5 | Vehicle |
| **21** | Mota Sosariya, Nani Vadal, Bhenkra | 1251.24 | 12.41 | Vehicle |
| **22** | Dedakadi, Gadhadka, Kedariya, Madhada, Nana Sosariya, Jhadakala | 561.77 | 16.12 | Vehicle |
| **23** | Mota Sosariya, Palaaniya,Ramgadh | 587.73 | 5.19 | Vehicle |

**Table 7. Details of vehicle/foot transects laid and area of Junagadh grasslands.**

| **Transect  No.** | **Transect Details** | **Area**  **(ha)** | **Length (km)** | **Transect Type** |
| --- | --- | --- | --- | --- |
| 1 | Vandarvad | 23.38 | 2.6 | Vehicle |
| 2 | Dadmadhar, Sardargadh, Sherdi, Utadi, Zizari, Sherdi Survey. No.292/1, Sardargadh, Mitadi, Navghanvalu Gana, Indrani Vidi Limbuda, Thapala, Gyasudikhanji anavar khaji Dadava, kodvav | 448.36 | 10 | Vehicle |
| 3 | Charakhada, Lakkaddhar, Kubaddhar | 421.17 | 7.3 | Vehicle |
| 4 | Motababra, Amridhar, Nanababra, Jalodhar, Lachhdi, Kathiyadhar | 818.45 | 26.6 | Vehicle |
| 5 | Dudhiya, Madhva Khageshri, Khatavadi, Dhuvada, Sukva, Dhuvada, Rajgadvari dhar, Dhuvada, Hajamdi, Dhuvada Dadadubha harjivanvadi dhar, Dosa Modhaveli, Khageshri, Charavado Bhagh, Dhuvada | 2181.02 | 34.2 | Vehicle |
| 6 | Sendarda, Madhda Pati, Ajab | 145.79 | 7 | Vehicle |
| 7 | Pandava, Inaj | 80.8 | 4.5 | Vehicle |
| 8 | Satalpur | 22.75 | 0.9 | Foot |
| 9 | Lamboda, Khodda, Masundridhar Shepa | 57.2 | 2.1 | Vehicle |
| 10 | Tadkapiliya | 76.76 | 1.9 | Vehicle |
| 11 | Kharabani vidi, Vadala, Margodungar, Devda, Roghda, Dhedhdidhar, Roghda, Maharajvadu, Helabeli, Bavdavadar | 807.56 | 11.8 | Vehicle |

**Table 8. Details of vehicle/foot transects laid and area of Bhavnagar grasslands.**

| **Transect  No.** | **Transect Details** | **Area**  **(ha)** | **Length (km)** | **Transect Type** |
| --- | --- | --- | --- | --- |
| 1 | Pavad, Kadtho Chhapro, Rojmal, Chavdidhar (Karamadiya) | 619.95 | 9.5 | Vehicle |
| 2 | Ukhrala, Chhaya, Khantdi | 204.29 | 2.4 | Vehicle |
| 3 | Juna Padar, Bhandar, Lakhanka | 86.2 | 2.6 | Vehicle |
| 4 | Bhavnagar Victoria Park | 202 | 6.8 | Vehicle |
| 5 | Piprala, Juna Sarod | 920.95 | 7.7 | Vehicle |
| 6 | Thala, Bhankhal, Sarkadiya Dhar | 408.61 | 7.2 | Vehicle |
| 7 | Chorvadla, Ishvariya, Pipardi | 1000.34 | 9.1 | Vehicle |
| 8 | Amargadh, Pipradi | 122.74 | 0.9 | Vehicle |
| 9 | Ranigala, Karjala, Kadiyali, Bhayagalo | 1403.6 | 14.2 | Vehicle |
| 10 | Baddhiyo | 83.66 | 1.6 | Vehicle |
| 11 | Nani Rajasthali, Sanjansar, Medha | 918.27 | 14.7 | Vehicle |
| 12 | Anida | 910.84 | 11.4 | Vehicle |
| 13 | Gebar, Dholiyadhar | 727.67 | 5.9 | Vehicle |
| 14 | Hamirpara, Sankhadsar-2 | 107.35 | 3.8 | Vehicle |
| 15 | Navkukri, Sagada Bhandar | 290.25 | 3.7 | Foot |
| 16 | Moda | 44.25 | 2 | Vehicle |
| 17 | Dhankaniya, Sarve Pati, Saliya, Khokhai, Sherthadi, Shethali Gundadu | 584.64 | 12 | Vehicle |
| 18 | Rohishala, Navaniya | 68.34 | 3.6 | Vehicle |
| 19 | Gadhda, Vavdi, Khopda, Holya, Mandavdhar, Nana Umarda | 362.68 | 14.3 | Vehicle |

**Table 9. Details of vehicle/foot transects laid in Coastal areas in the landscape.**

| **Transect  No.** | **Transect Details** | **Area**  **(ha)** | **Length (km)** | **Transect**  **Type** |
| --- | --- | --- | --- | --- |
| 1 | Chhara | 228.33 | 3.4 | Foot |
| 2 | Sarkhadi to Velan | 359.1 | 2.2 | Vehicle |
| 3 | Simar | 56.17 | 4 | Foot |
| 4 | Senjaliya | 283.28 | 0.4 | Foot |
| 5 | Nabandar-Nandan-Naliya Mandri | 406.31 | 3.6 | Vehicle |
| 6 | Sheriyaj bara to Mangrol Bandar | 126.81 | 2.9 | Foot |
| 7 | Mangrol Bandar to Mukutupur | 33.84 | 5 | Foot |
| 8 | Rahij to Loej | 12.87 | 3.2 | Foot |
| 9 | Loej to Shil | 81.5 | 2.8 | Foot |
| 10 | Shil to Sangvada | 234.02 | 2.2 | Foot |
| 11 | Chorvad Choki to Jhunjarpur | 48.56 | 5.8 | Foot |
| 12 | Jhunjarpur to Khambhadiya Border | 55.14 | 2.3 | Foot |
| 13 | Khambhadiya to Khodda | 39.44 | 2.2 | Foot |
| 14 | Nayabara to Sheriyaj Bara | 268.98 | 4.5 | Foot |
| 15 | Hathab to Kodiyak | 108.56 | 4.7 | Foot |
| 16 | Hathab to Khadsaliya | 14.59 | 1.2 | Foot |
| 17 | Chopra | 20.23 | 4.1 | Foot |
| 18 | Padri Gohil | 40.47 | 3.6 | Foot |
| 19 | Tarsara | 42.49 | 3.5 | Foot |
| 20 | Sartanpar | 56.33 | 5.2 | Foot |
| 21 | Jhanjmer | 26.3 | 2.3 | Foot |
| 22 | Madhuvan to methla Border | 33.3 | 8.1 | Foot |
| 23 | Methda to Uncha nicha Kotda | 742.9 | 6.6 | Foot |
| 24 | Uncha Kotda | 82.95 | 3.3 | Foot |
| 25 | Nicha Kotda | 32.37 | 2.4 | Foot |
| 26 | Dayal to Kalsar | 121.41 | 3.8 | Foot |
| 27 | Vaghngar to Katpar City | 52.6 | 2 | Foot |
| 28 | Khared-Gujarada-Dudheri-Doliya | 101.9 | 7.6 | Vehicle |
| 29 | Vadodra Dodiya to Veraval | 150.34 | 4.3 | Foot |
| 30 | Adri to Navapara | 49.06 | 4.2 | Foot |
| 31 | Dari | 139.99 | 5.3 | Foot |
| 32 | Lati | 108.52 | 3.9 | Foot |
| 33 | Kadvar | 53.93 | 3.5 | Foot |
| 34 | Sutrapada | 237.52 | 3.6 | Foot |
| 35 | Dhamlej-1 | 202.62 | 5.4 | Foot |
| 36 | Dhamlej-2 | 102.71 | 4.5 | Foot |
| 37 | Bherai | 103 | 2.4 | Foot |
| 38 | Kovaya | 206 | 3 | Foot |
| 39 | Patva | 94 | 5 | Foot |
| 40 | Babarkot | 354 | 1.9 | Foot |

*****
