# Supplementary material for: The semi-arid ecosystem of Asiatic Lion Landscape in Saurashtra, Gujarat: Population density, biomass and conservation of nine wild prey species
Source: PLoS One. 2023 Sep 28;18(9):e0292048. doi: 10.1371/journal.pone.0292048 (PMC10538734; doi:10.1371/journal.pone.0292048)
Supplement: S3 File — (DOCX) [file pone.0292048.s003.docx]

**Number of groups encountered during the sampling period at different study sites.**

| **Species** | **Number of groups encountered** | | | | | | | |
| --- | --- | --- | --- | --- | --- | --- | --- | --- |
|  | **Gir** | **Mitiyala** | **Paniya** | **Girnar** | **Gir grasslands** | **Junagadh grasslands** | **Bhavnagar grasslands** | **Coastal forests** |
| **Spotted deer** | 1236 | 118 | 34 | 189 | 460 | 78 | 56 | 69 |
| **Sambar** | 230 | 3 | 4 | 72 | 10 | 1 | 0 | 0 |
| **Blue bull** | 74 | 18 | 2 | 17 | 301 | 230 | 395 | 389 |
| **Four-horned antelope** | 6 | 0 | 1 | 2 | 6 | 1 | 0 | 0 |
| **Indian gazelle** | 23 | 0 | 4 | 0 | 55 | 8 | 159 | 0 |
| **Blackbuck** | 0 | 0 | 0 | 0 | 8 | 53 | 9 | 0 |
| **Wild pig** | 67 | 4 | 6 | 8 | 75 | 39 | 19 | 93 |
| **Hanuman langur** | 400 | 0 | 5 | 38 | 22 | 0 | 0 | 0 |
| **Indian peafowl** | 1363 | 64 | 60 | 262 | 511 | 190 | 287 | 287 |
