# Supplementary material for: The semi-arid ecosystem of Asiatic Lion Landscape in Saurashtra, Gujarat: Population density, biomass and conservation of nine wild prey species
Source: PLoS One. 2023 Sep 28;18(9):e0292048. doi: 10.1371/journal.pone.0292048 (PMC10538734; doi:10.1371/journal.pone.0292048)
Supplement: S4 File — (DOCX) [file pone.0292048.s004.docx]

**Detection probability and distance data for different wild prey species in different study sites in Asiatic Lion Landscape, Gujarat, India.**


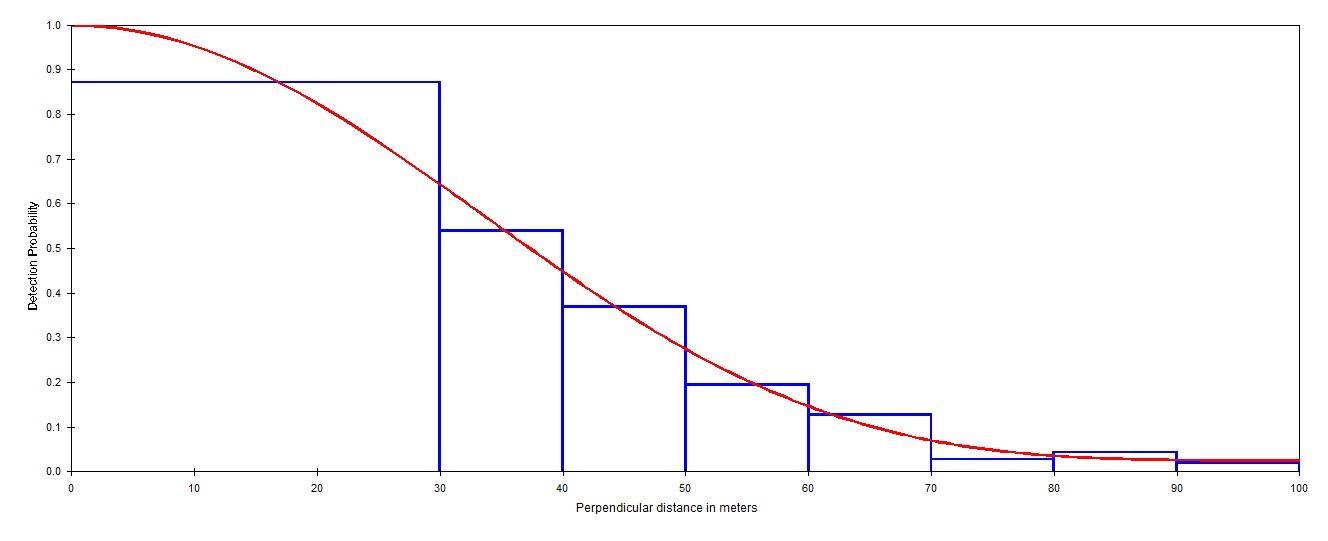


**Fig 1. Detection probability and distance data for spotted deer truncated at 100 m, and fitted with the half normal model in Gir National Park and Wildlife Sanctuary.**


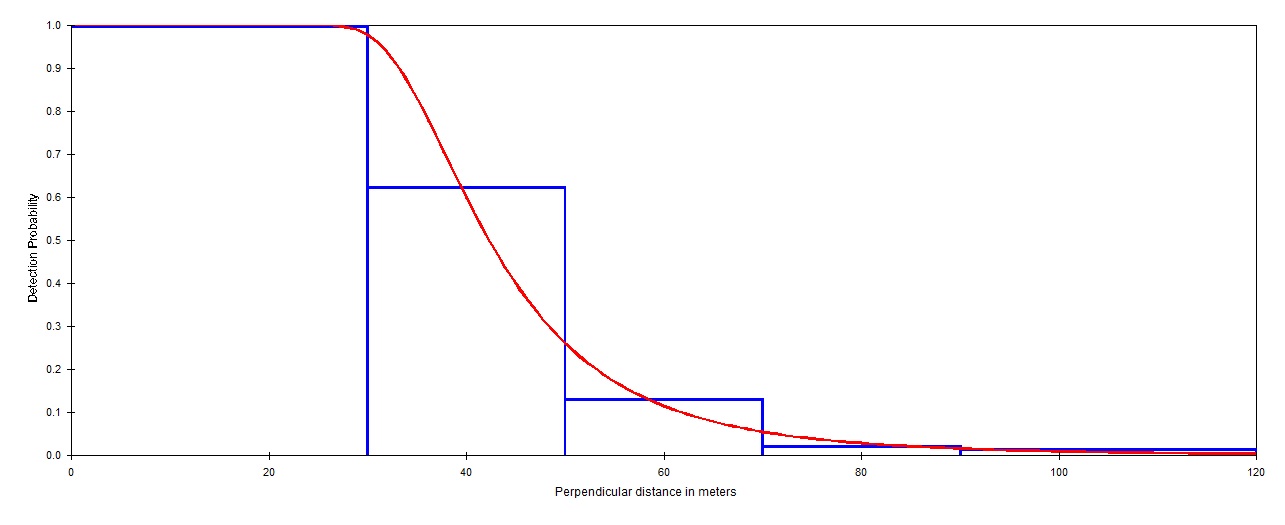


**Fig 2. Detection probability and distance data for sambar truncated at 120 m, and fitted with the hazard rate model in Gir National Park and Wildlife Sanctuary.**


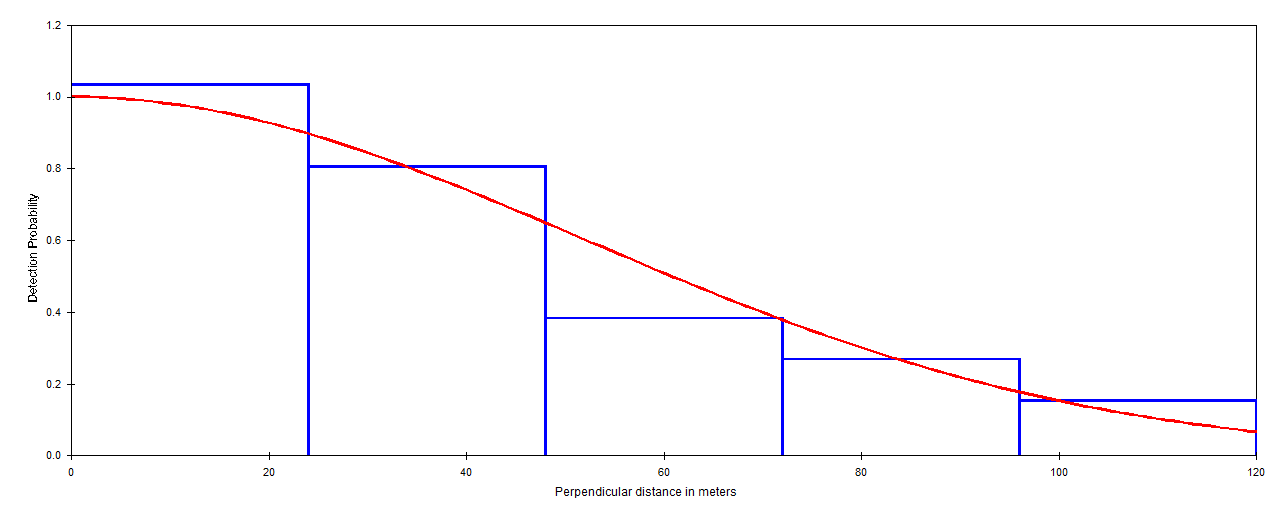


**Fig 3. Detection probability and distance data for blue bull truncated at 120 m, and fitted with the hazard rate model in Gir National Park and Wildlife Sanctuary.**


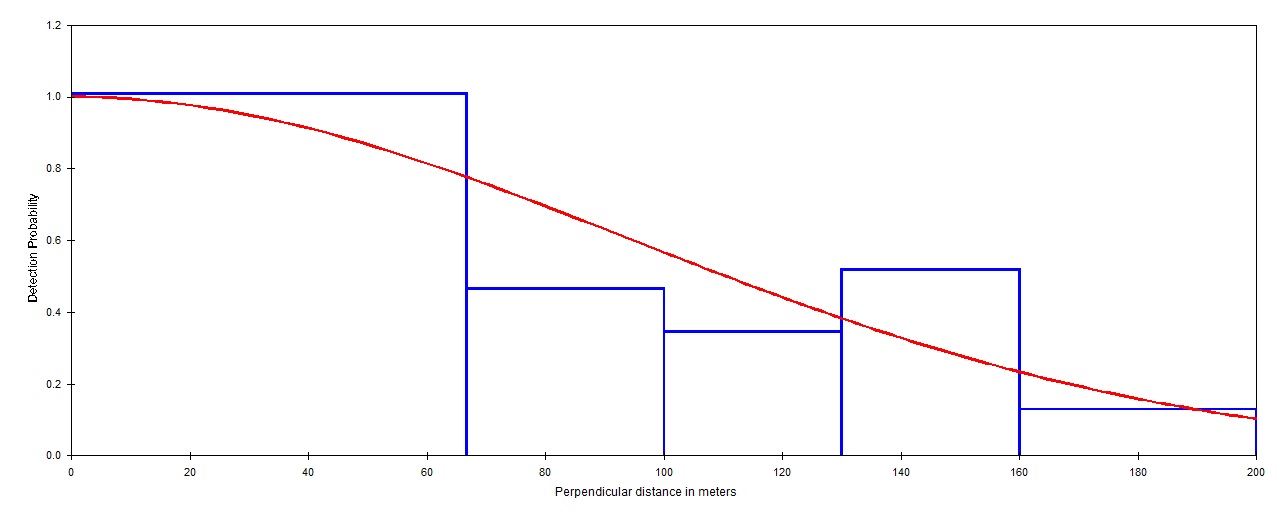


**Fig 4. Detection probability and distance data for Indian gazelle truncated at 200 m, and fitted with the hazard rate model in Gir National Park and Wildlife Sanctuary.**


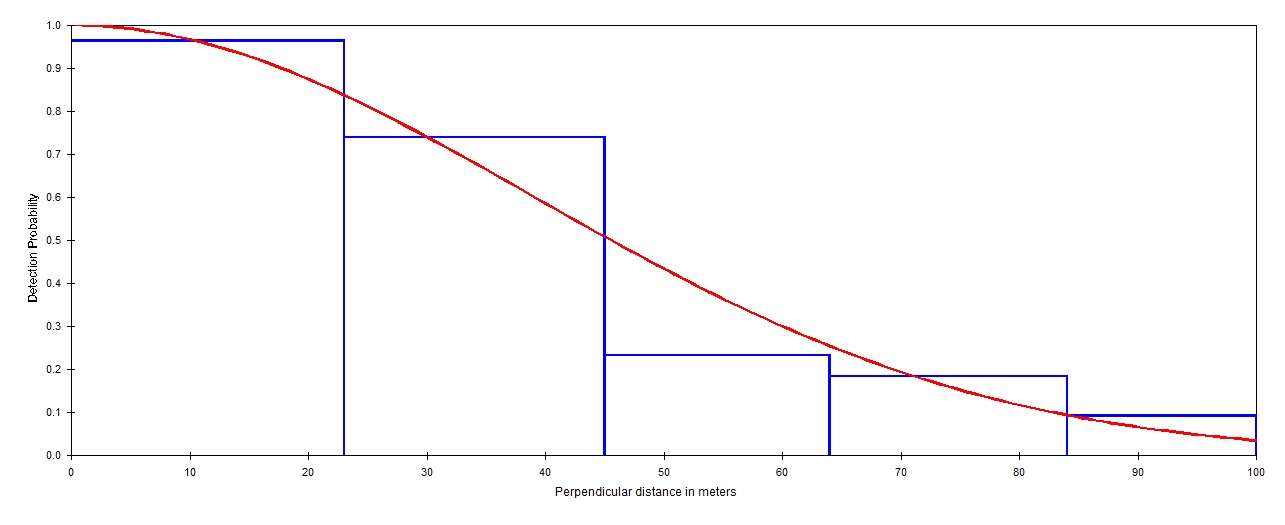


**Fig 5. Detection probability and distance data for wild pig truncated at 100 m, and fitted with the hazard rate model in Gir National Park and Wildlife Sanctuary.**


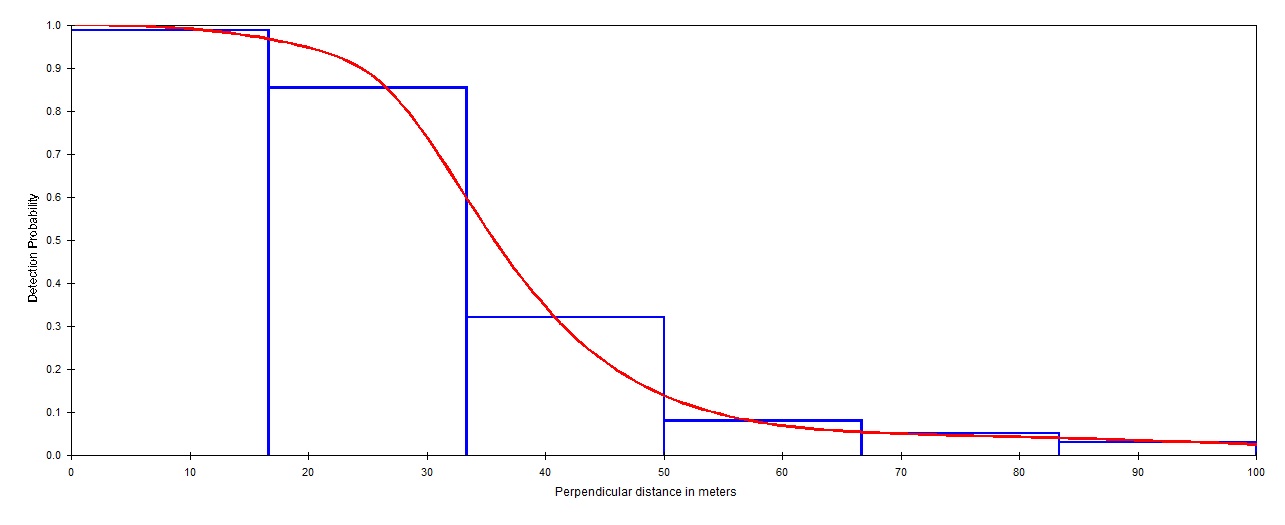


**Fig 6. Detection probability and distance data for Indian peafowl truncated at 100 m, and fitted with the hazard rate model in Gir National Park and Wildlife Sanctuary.**


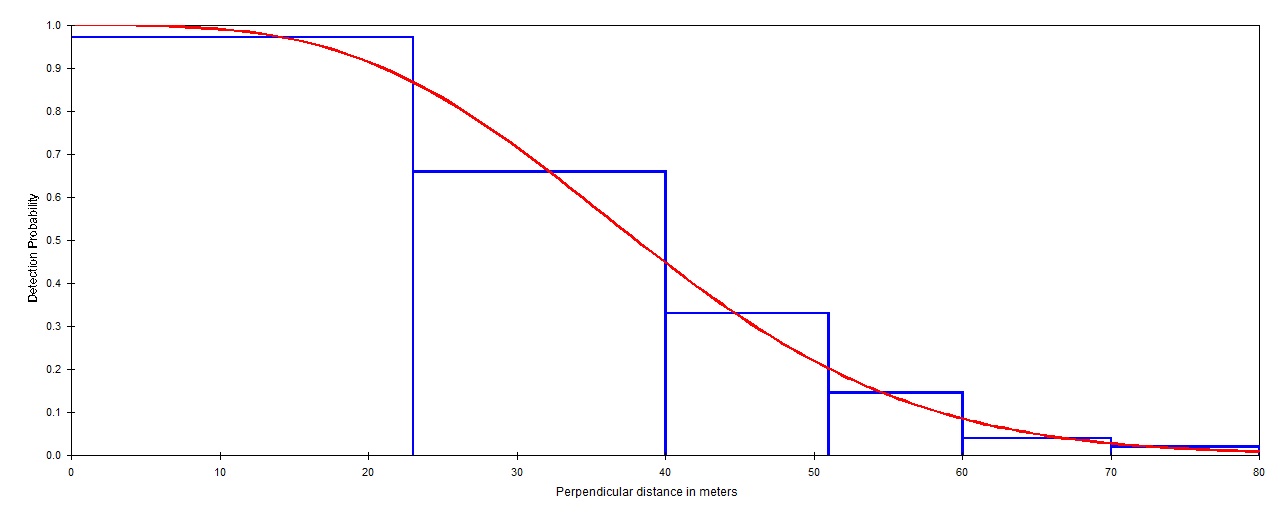


**Fig 7. Detection probability and distance data for Hanuman langur truncated at 80 m, and fitted with the hazard rate model in Gir National Park and Wildlife Sanctuary.**


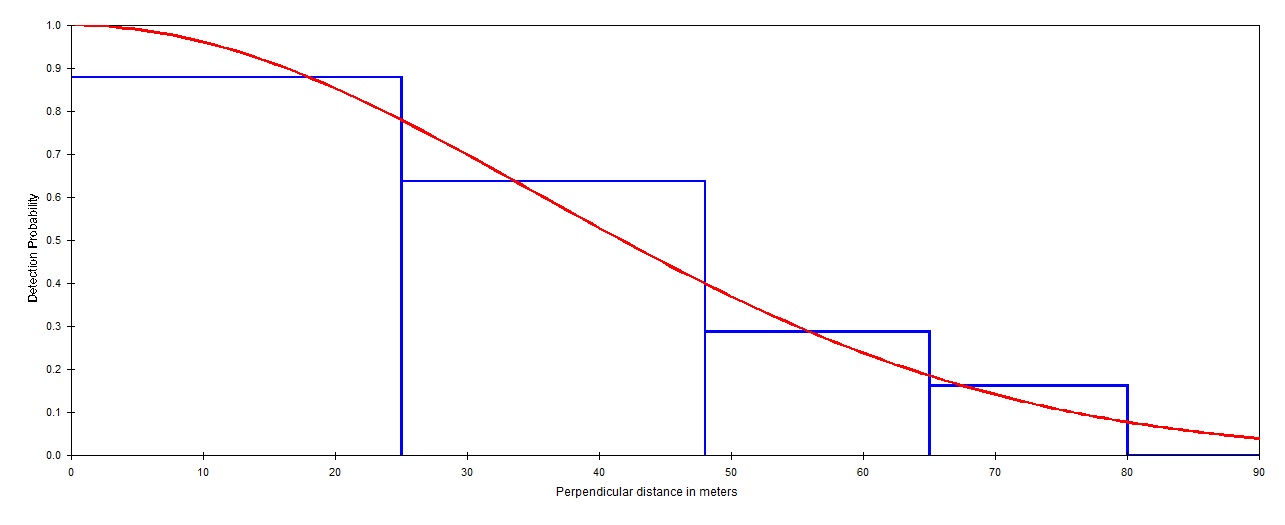


**Fig 8. Detection probability and distance data for Spotted deer truncated at 90 m, and fitted with the hazard rate model in Mitiyala Wildlife Sanctuary.**


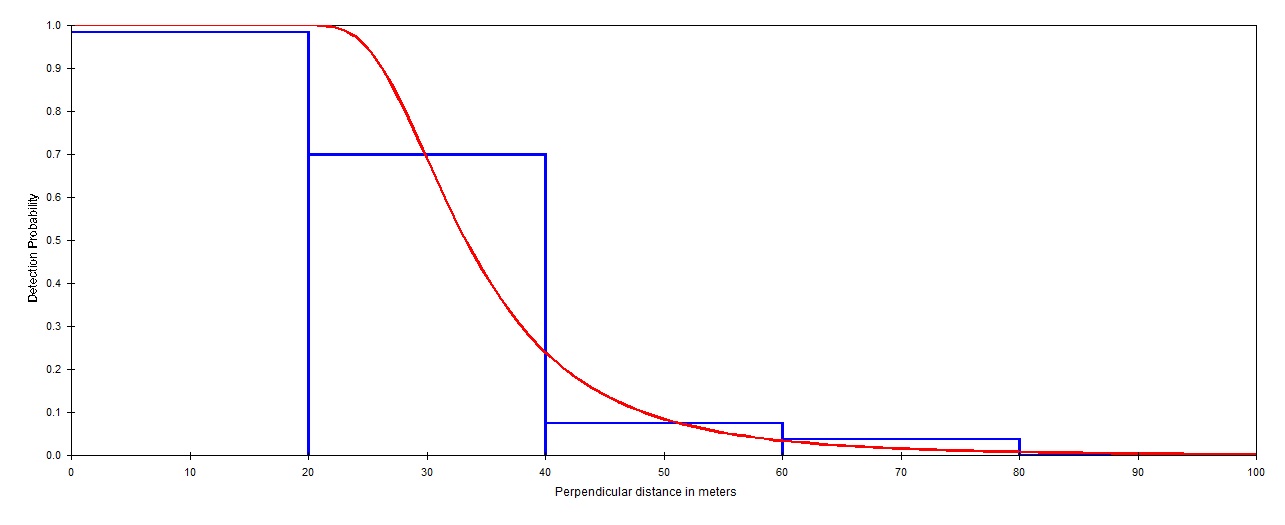


**Fig 9. Detection probability and distance data for blue bull truncated at 100 m, and fitted with the uniform model in Mitiyala Wildlife Sanctuary.**


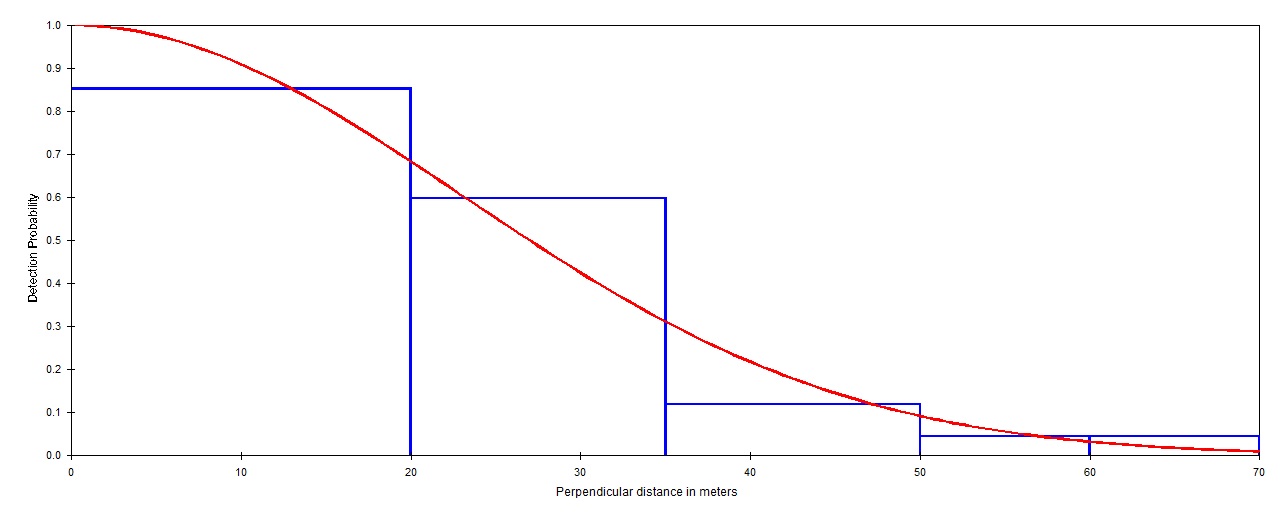


**Fig 10. Detection probability and distance data for Indian peafowl truncated at 70 m, and fitted with the Half normal in Mitiyala Wildlife Sanctuary.**


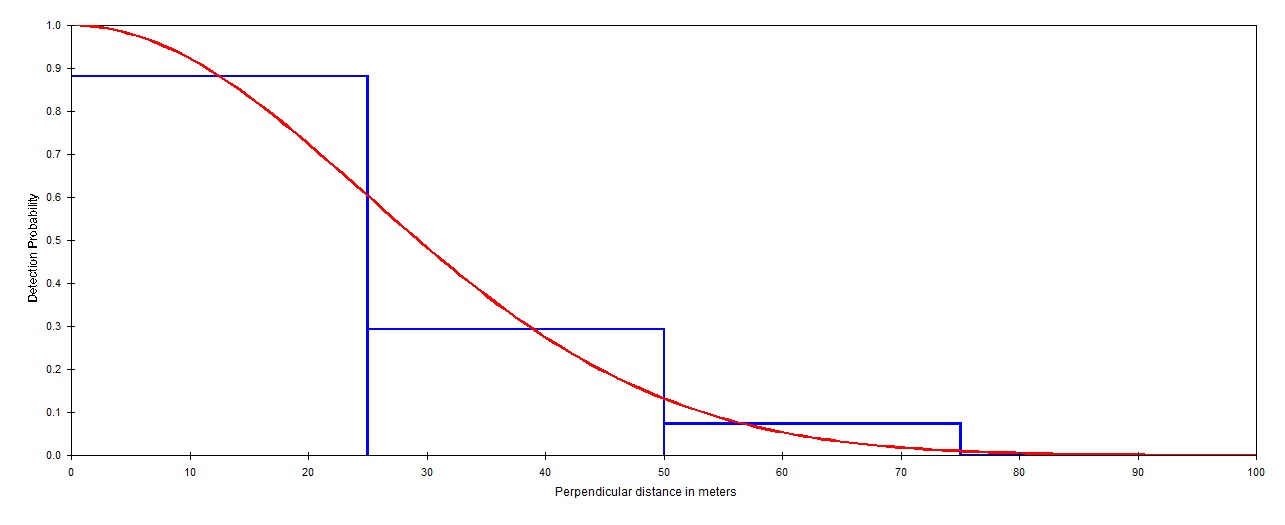


**Fig 11. Detection probability and distance data for Spotted deer truncated at 70 m, and fitted with the half normal in Paniya Wildlife Sanctuary.**


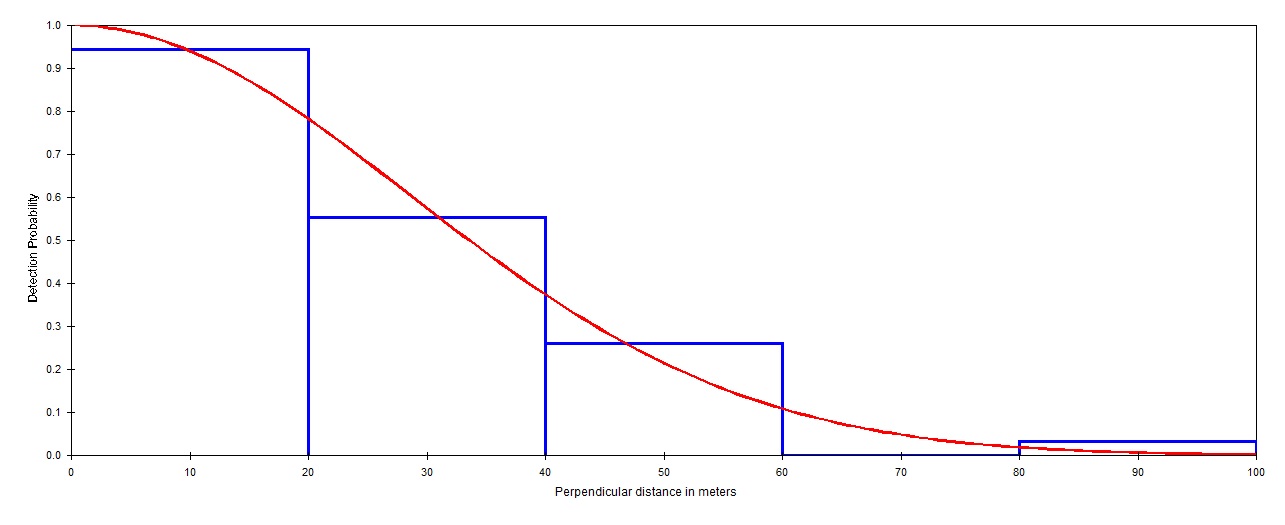


**Fig 12. Detection probability and distance data for Indian peafowl truncated at 100 m, and fitted with the Half normal in Paniya Wildlife Sanctuary.**


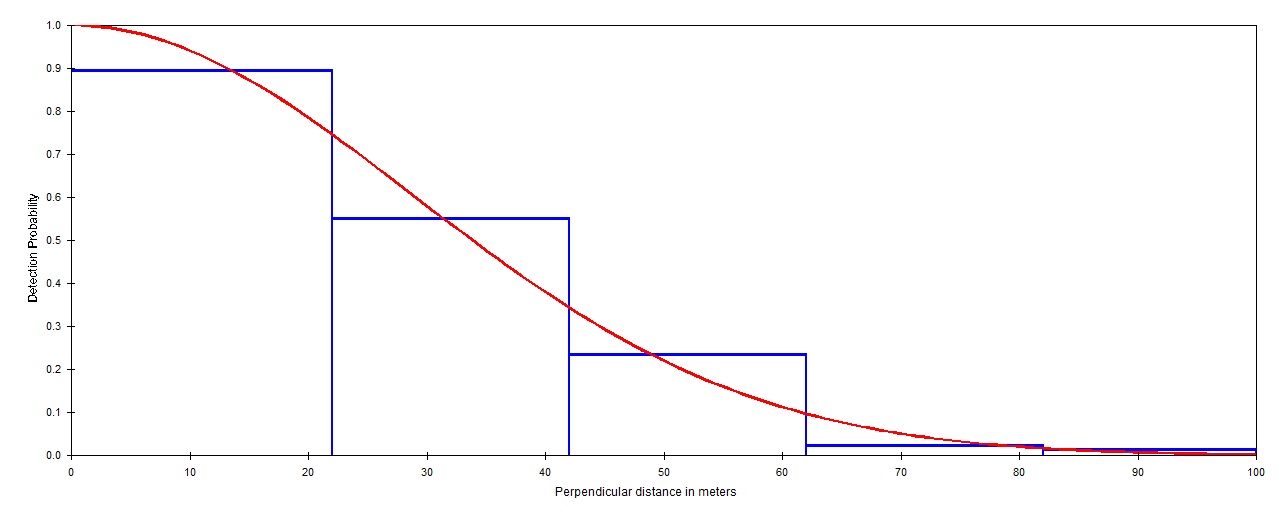


**Fig 13. Detection probability and distance data for Spotted deer truncated at 100 m, and fitted with the Half normal in Girnar Wildlife Sanctuary.**


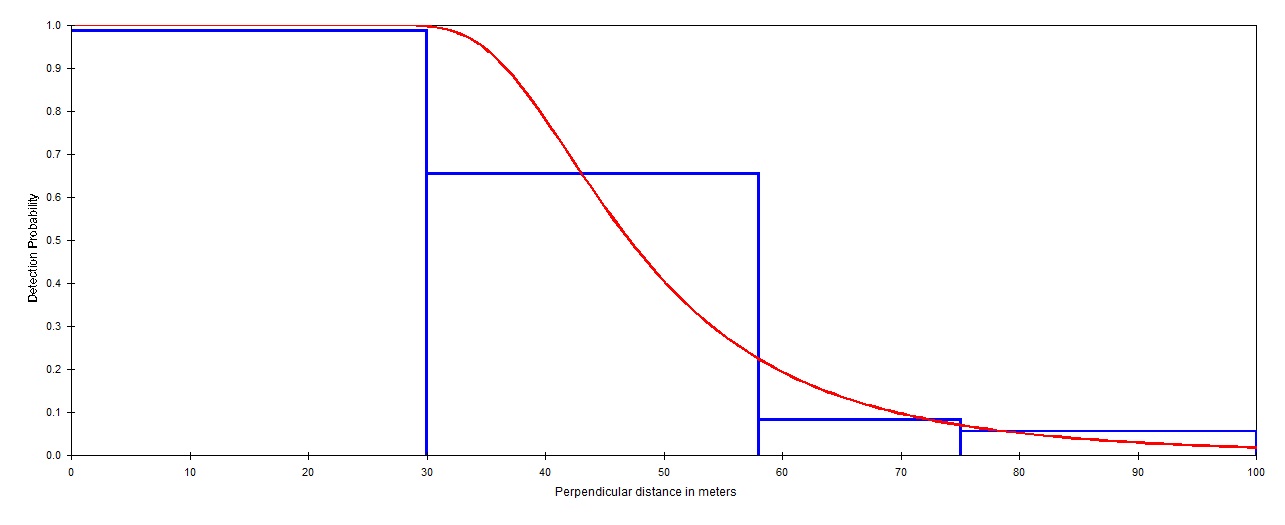


**Fig 14. Detection probability and distance data for Sambar truncated at 100 m, and fitted with the hazard rate model in Girnar Wildlife Sanctuary.**


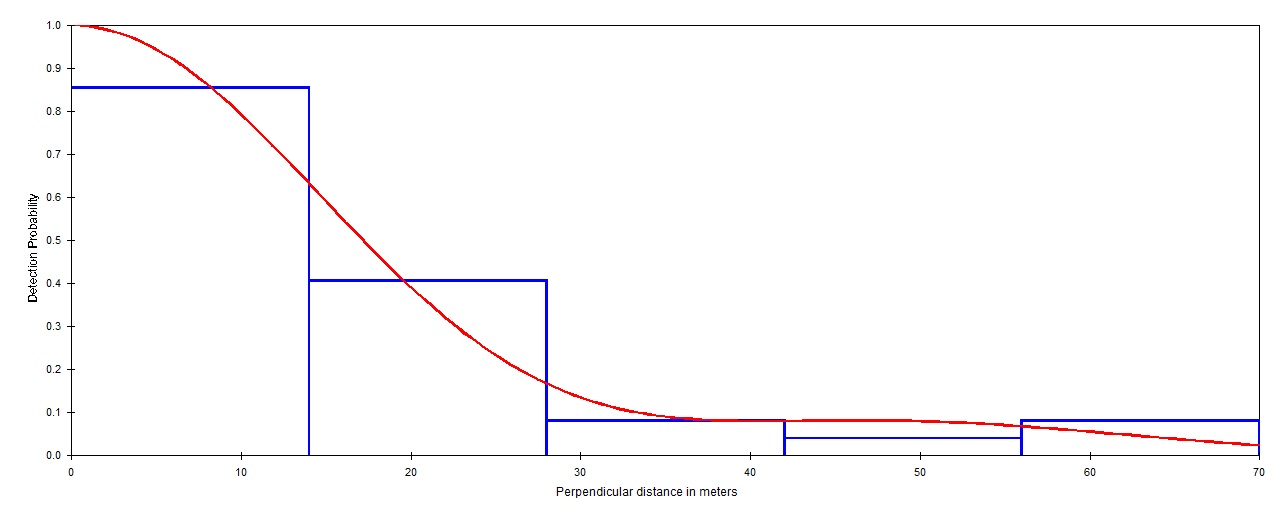


**Fig 15. Detection probability and distance data for Hanuman langur truncated at 70 m, and fitted with the half normal in Girnar Wildlife Sanctuary.**


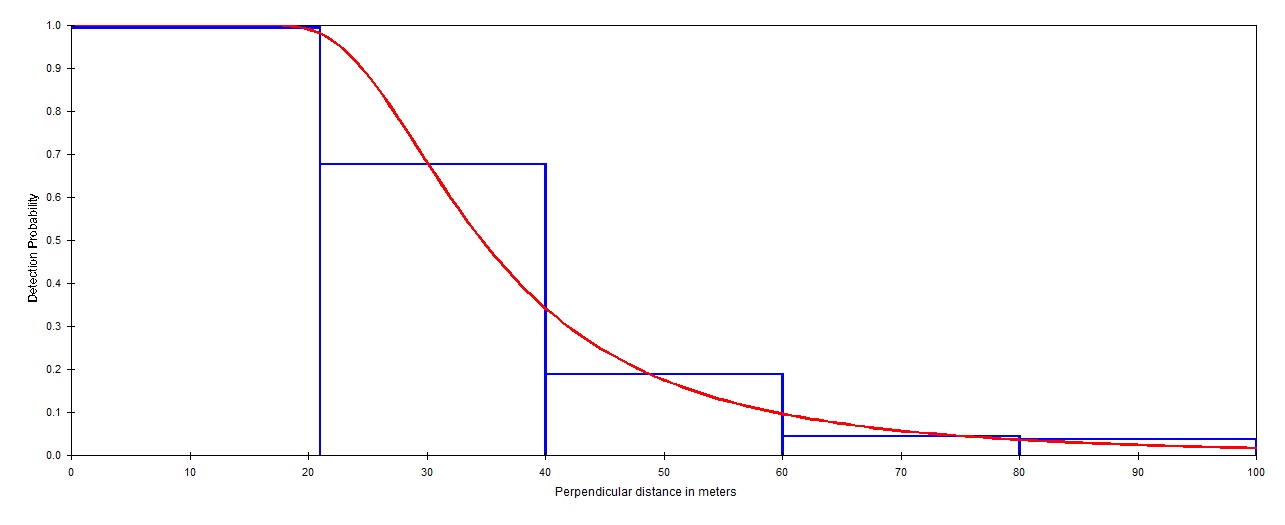


**Fig 16. Detection probability and distance data for Indian peafowl truncated at 100 m, and fitted with the half normal in Girnar Wildlife Sanctuary.**


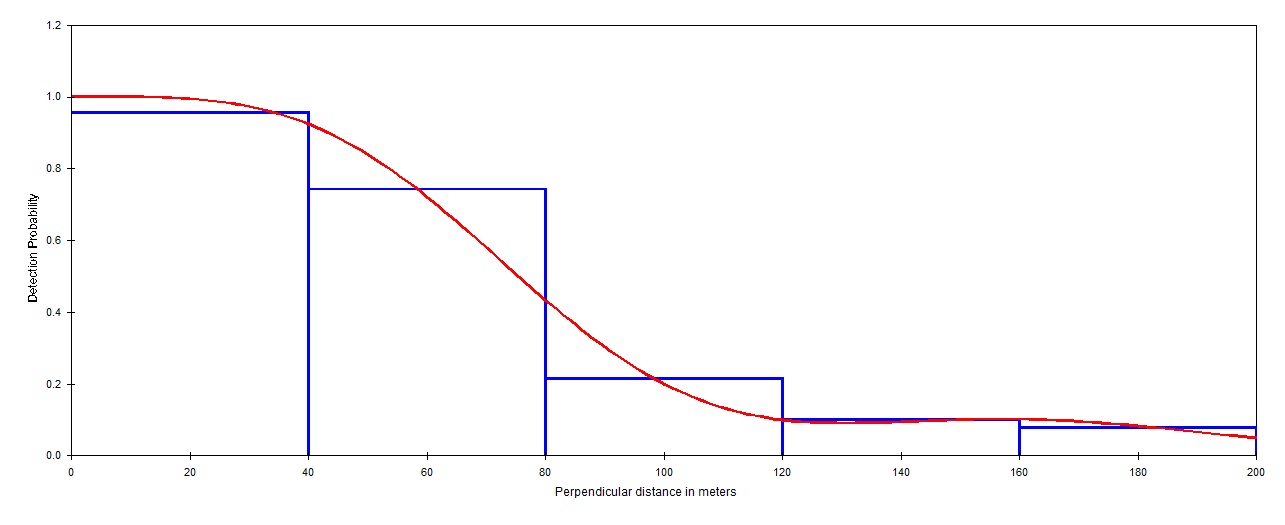


**Fig 17. Detection probability and distance data for Spotted deer truncated at 200 m, and fitted with the hazard rate model in Gir grasslands.**


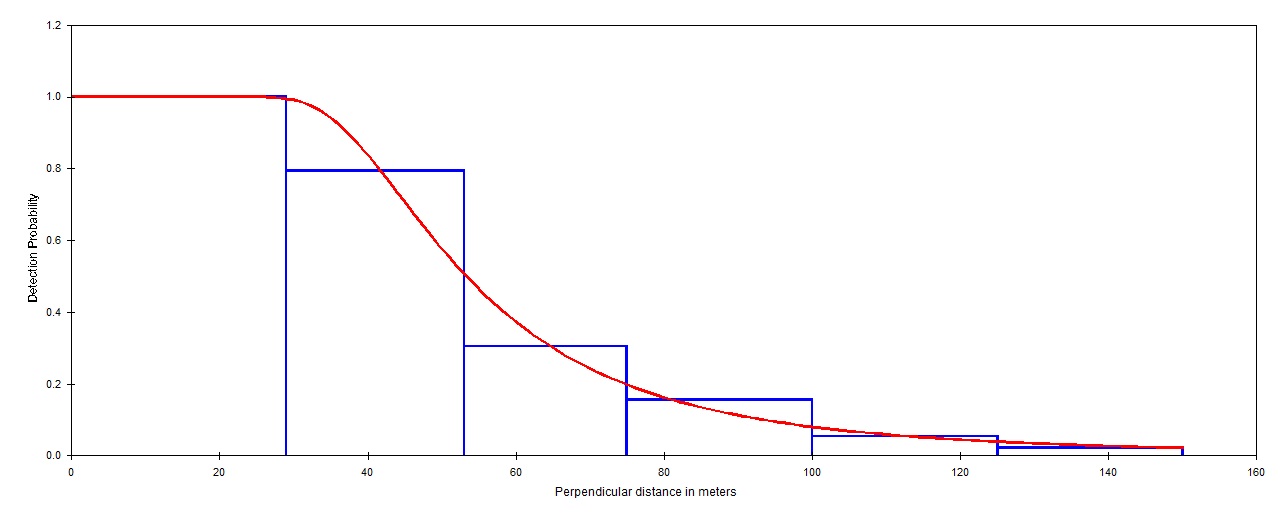


**Fig 18. Detection probability and distance data for blue bull truncated at 160 m, and fitted with the hazard rate model in Gir grasslands.**


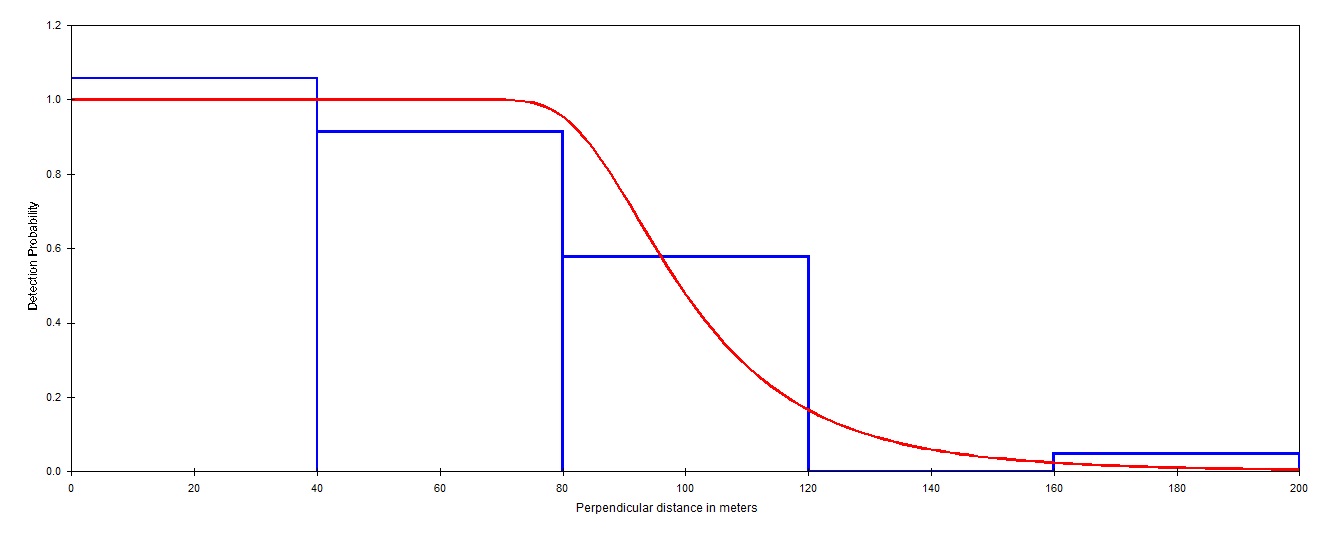


**Fig 19. Detection probability and distance data for Indian gazelle truncated at 200 m, and fitted with the hazard rate model in Gir grasslands.**


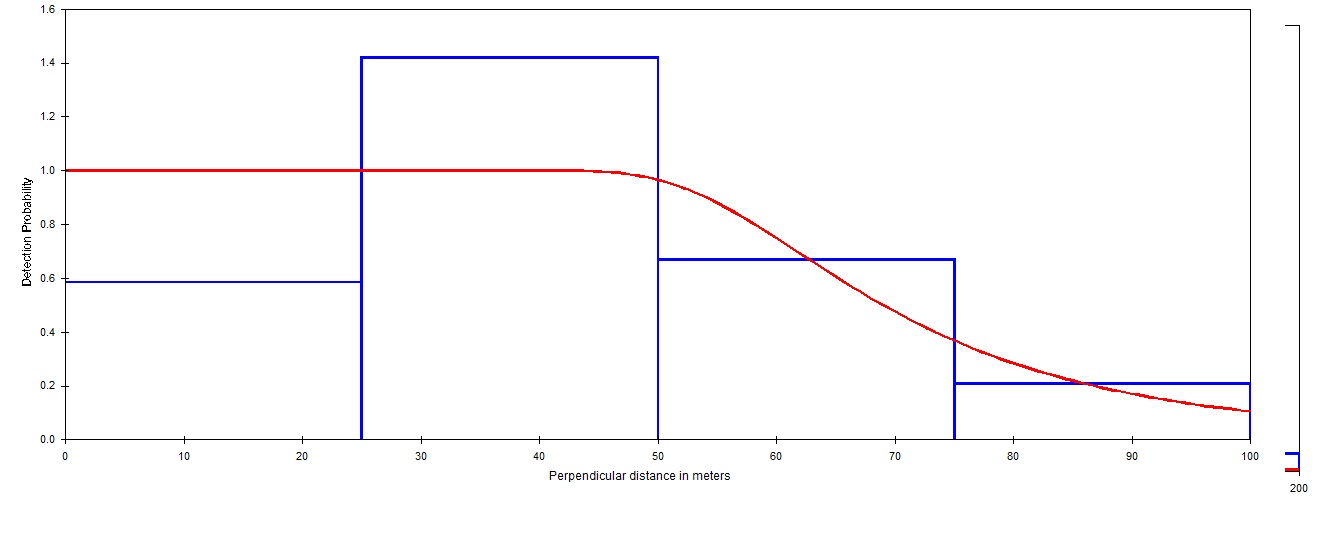


**Fig 20. Detection probability and distance data for wild pig truncated at 100 m, and fitted with the hazard rate model in Gir grasslands.**


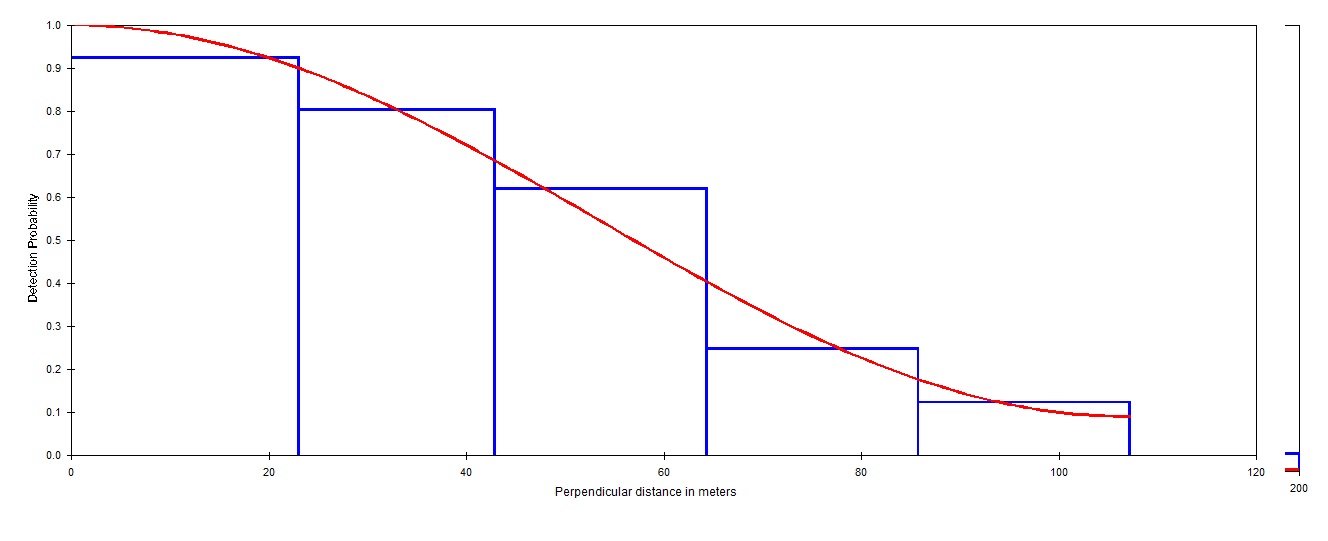


**Fig 21. Detection probability and distance data for Hanuman langur truncated at 120 m, and fitted with the uniform model in Gir grasslands.**


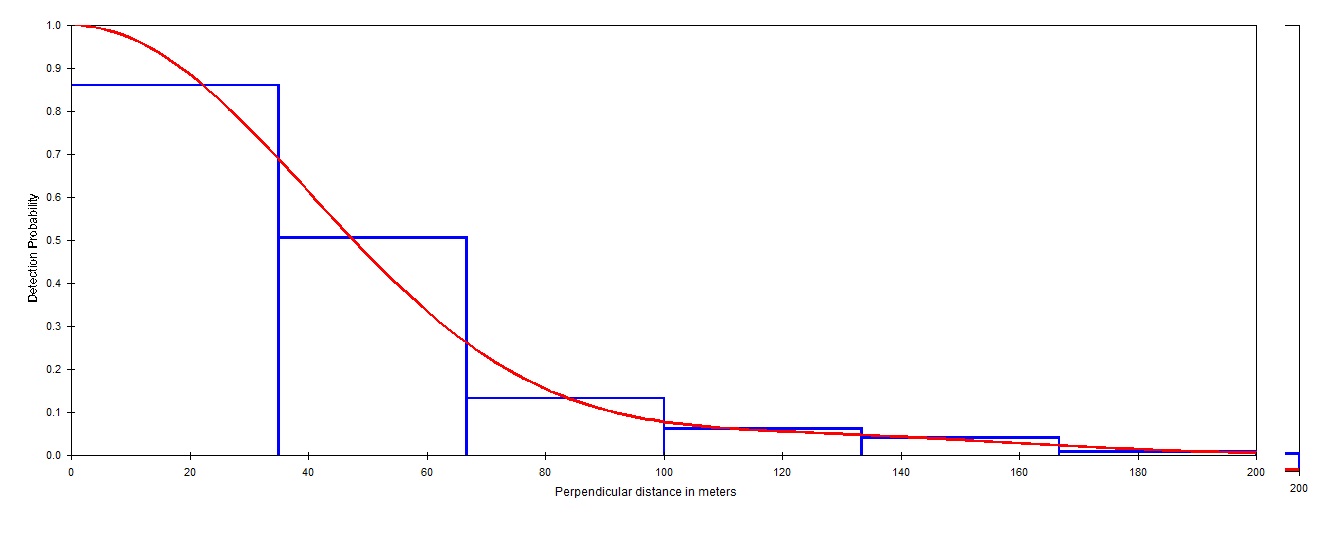


**Fig 22. Detection probability and distance data for Indian peafowl truncated at 200 m, and fitted with the half normal model in Gir grasslands.**


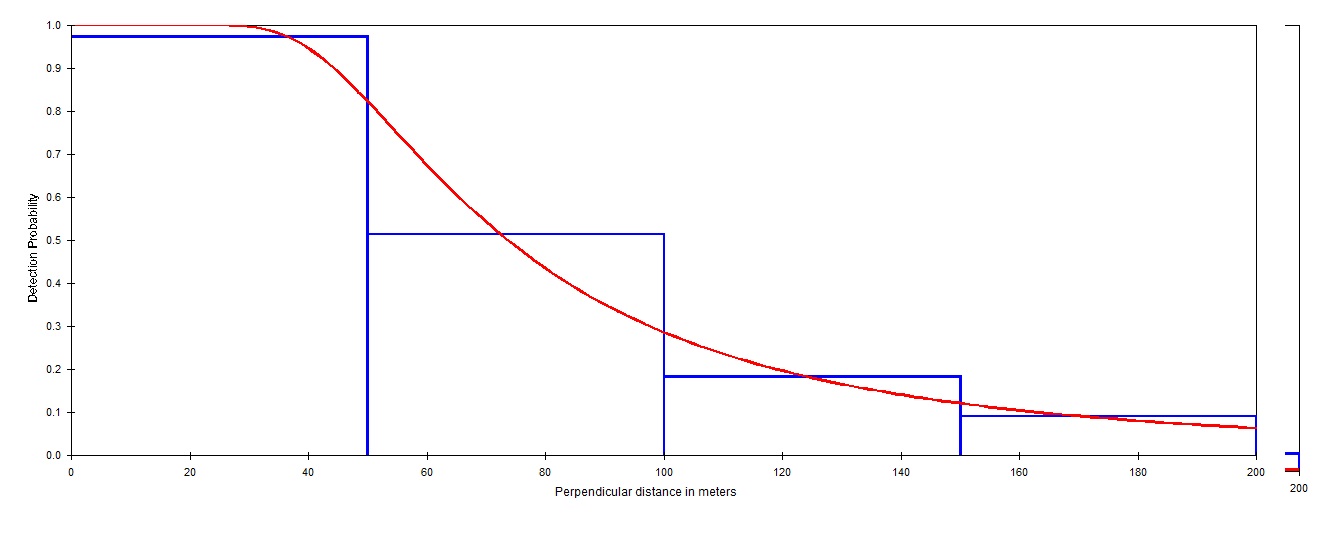


**Fig 23. Detection probability and distance data for Spotted deer truncated at 200 m, and fitted with the hazard rate model in Junagadh grasslands.**


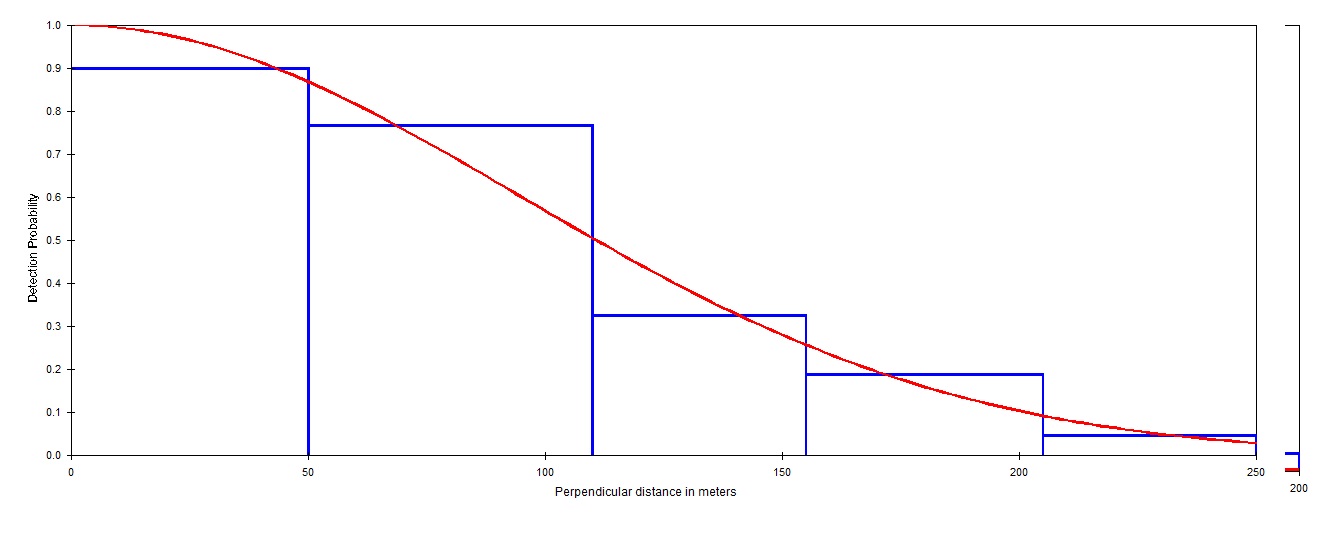


**Fig 24. Detection probability and distance data for blue bull truncated at 250 m, and fitted with the half normal model in Junagadh grasslands.**


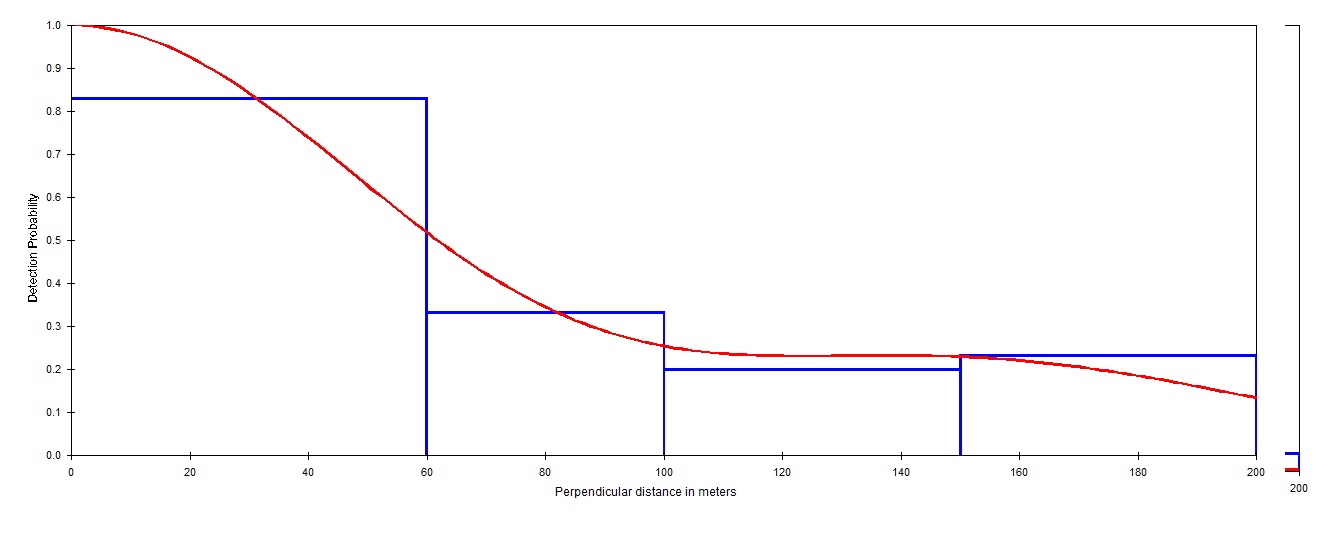


**Fig 25. Detection probability and distance data for Blackbuck truncated at 200 m, and fitted with the hazard rate model in Junagadh grasslands.**


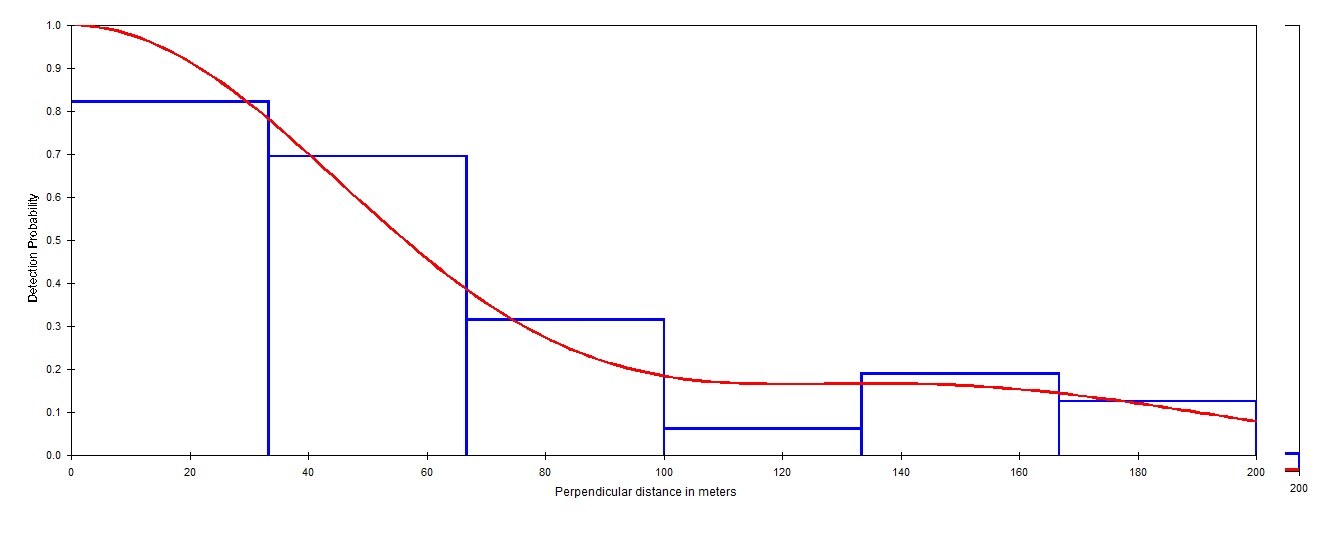


**Fig 26. Detection probability and distance data for Wild pig truncated at 200 m, and fitted with the half normal model in Junagadh grasslands.**


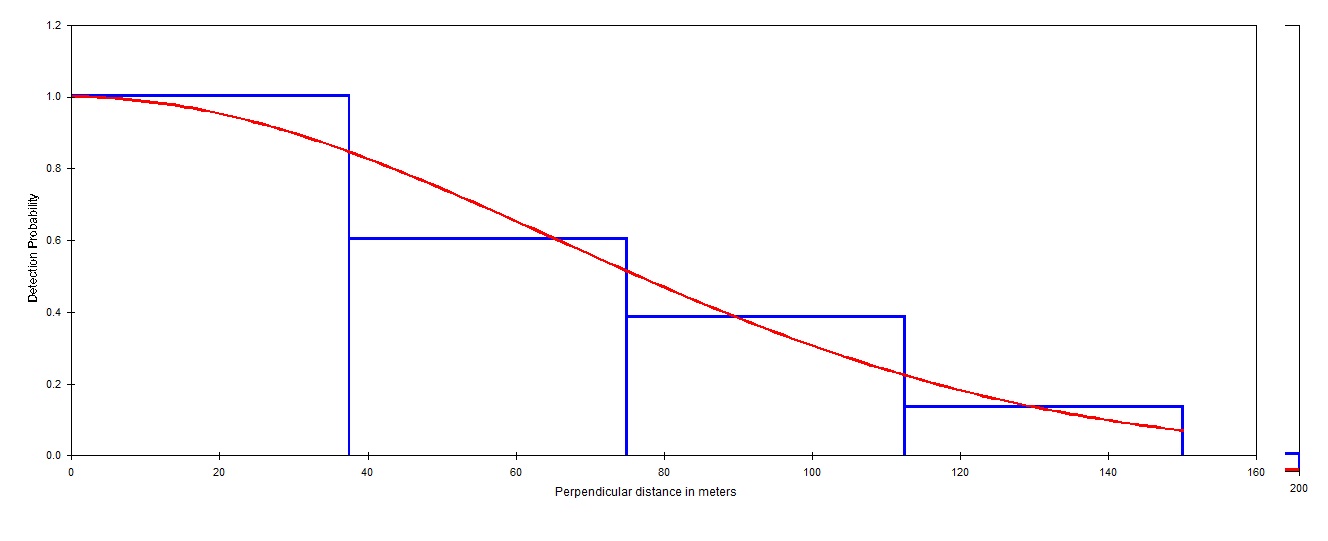


**Fig 27. Detection probability and distance data for Indian peafowl truncated at 160 m, and fitted with the half normal model in Junagadh grasslands.**


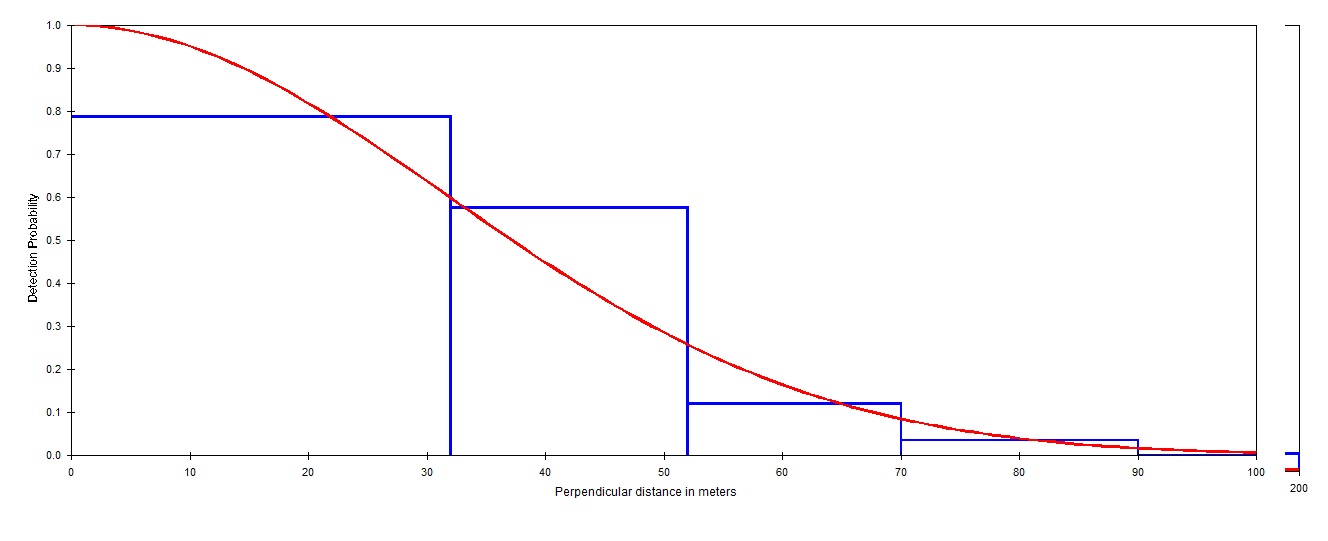


**Fig 28. Detection probability and distance data for Spotted deer truncated at 100 m, and fitted with the half normal model in Bhavnagar grasslands.**


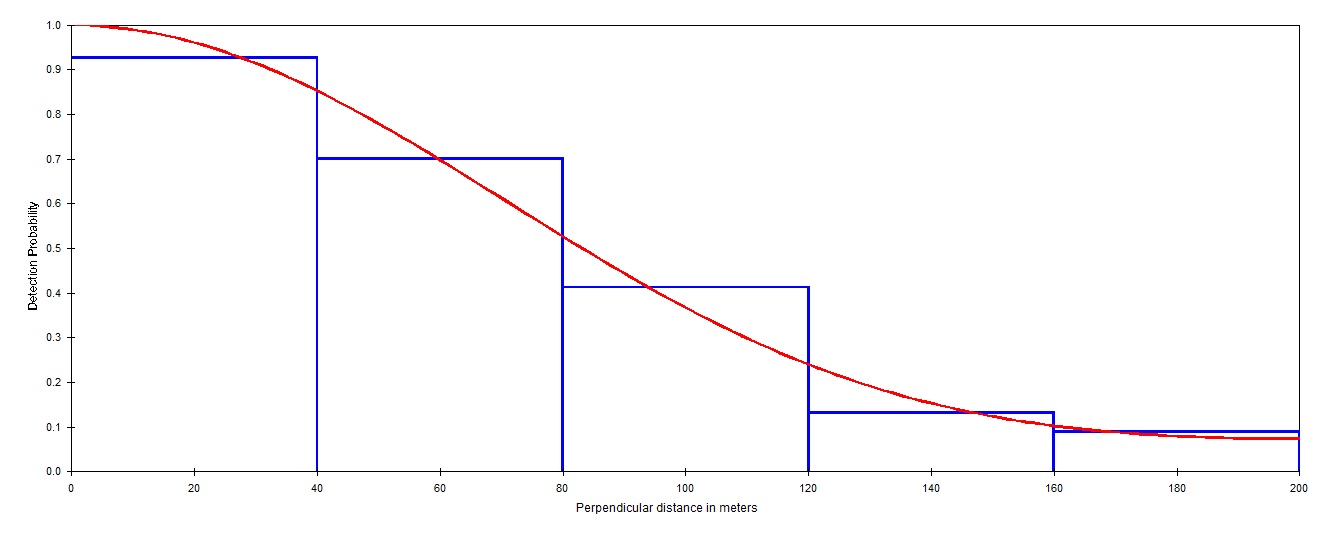


**Fig 29. Detection probability and distance data for blue bull truncated at 200 m, and fitted with the hazard rate model in Bhavnagar grasslands.**


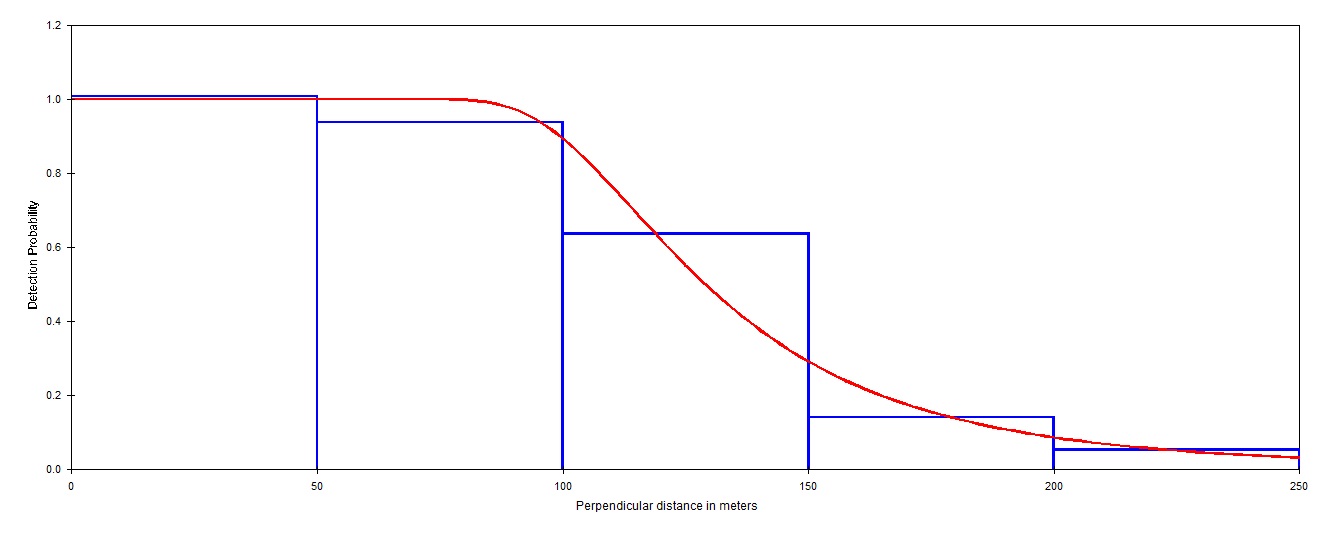


**Fig 30. Detection probability and distance data for Indian gazelle truncated at 250 m, and fitted with the hazard rate model in Bhavnagar grasslands.**


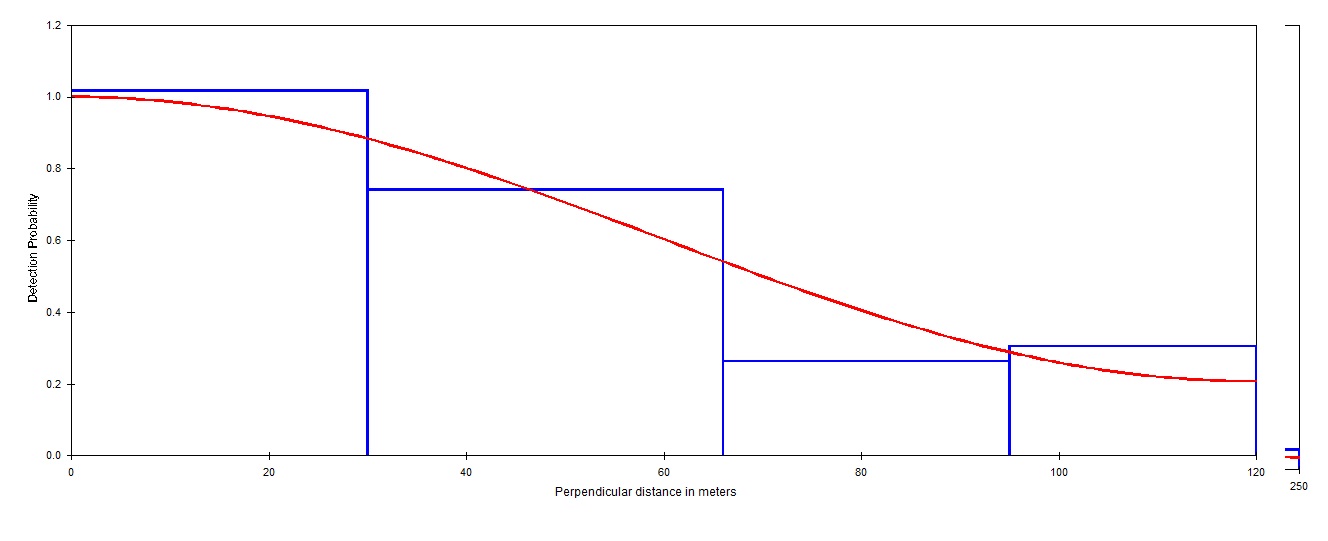


**Fig 31. Detection probability and distance data for Wild pig truncated at 120 m, and fitted with the uniform model in Bhavnagar grasslands.**


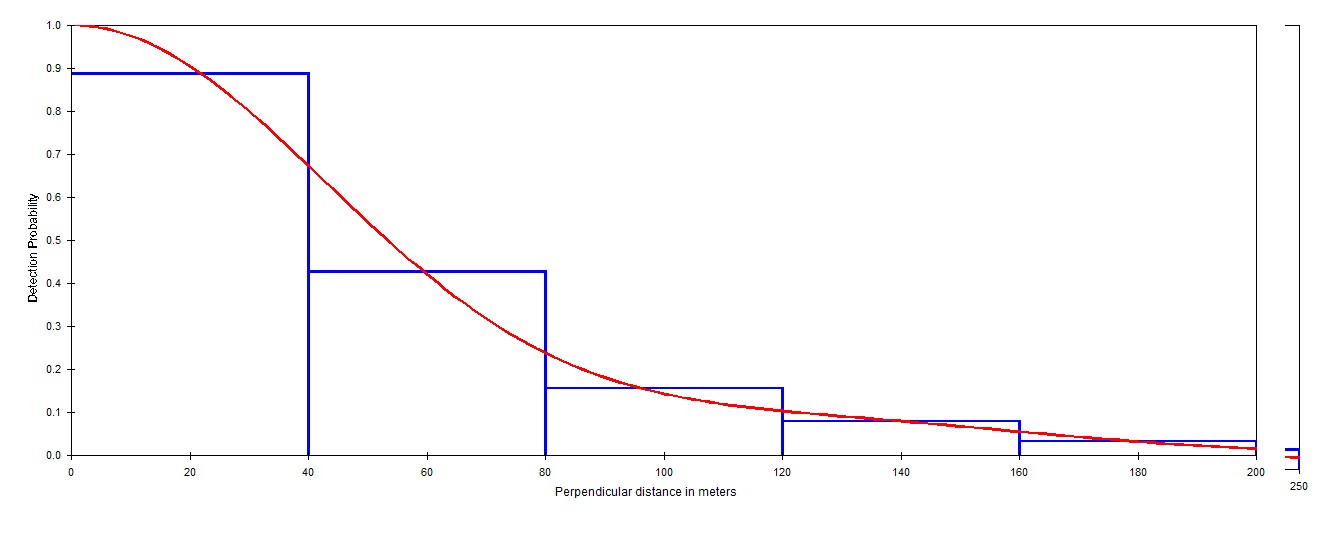


**Fig 32. Detection probability and distance data for Indian peafowl truncated at 200 m, and fitted with the half normal model in Bhavnagar grasslands.**


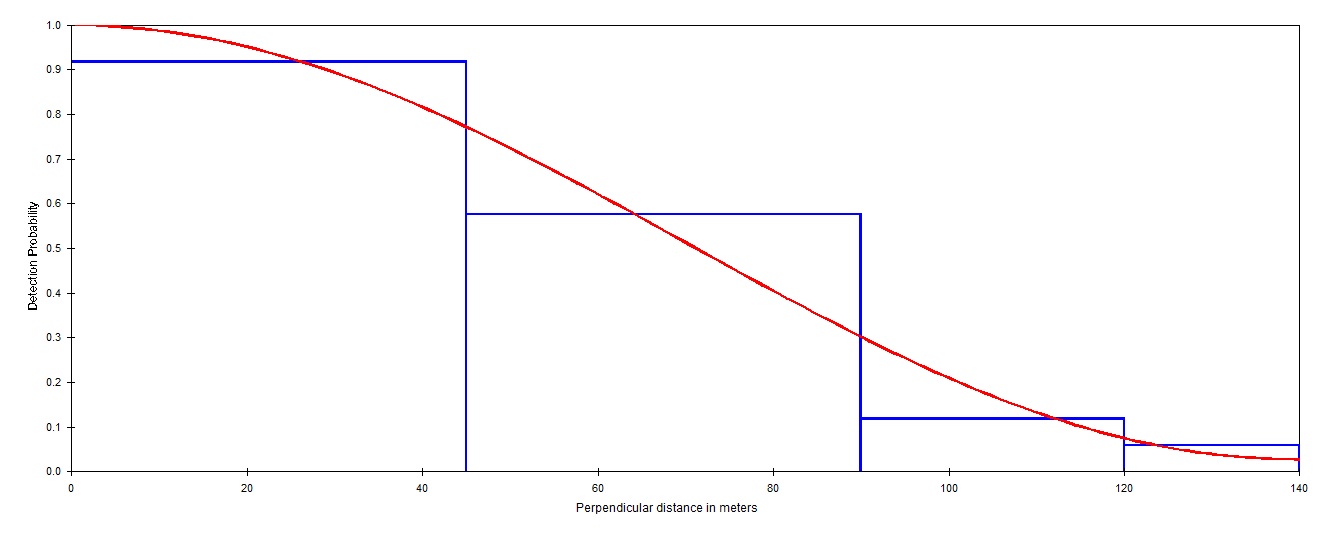


**Fig 33. Detection probability and distance data for Spotted deer truncated at 140 m, and fitted with the uniform model in Coastal forests.**


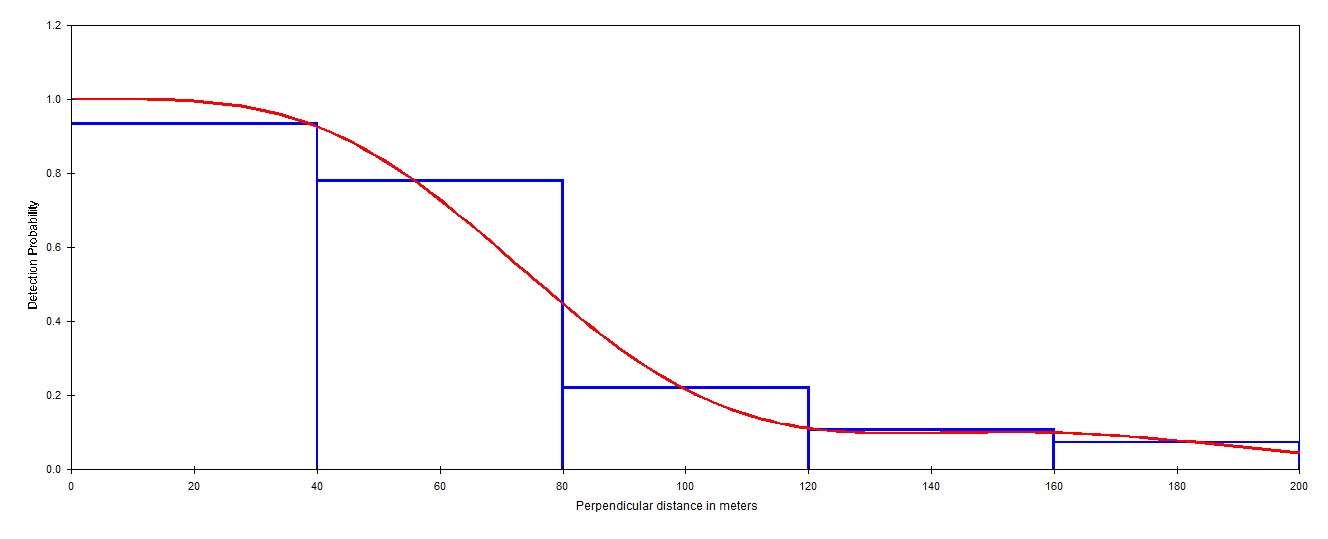


**Fig 34. Detection probability and distance data for blue bull truncated at 200 m, and fitted with the half normal model in Coastal forests.**


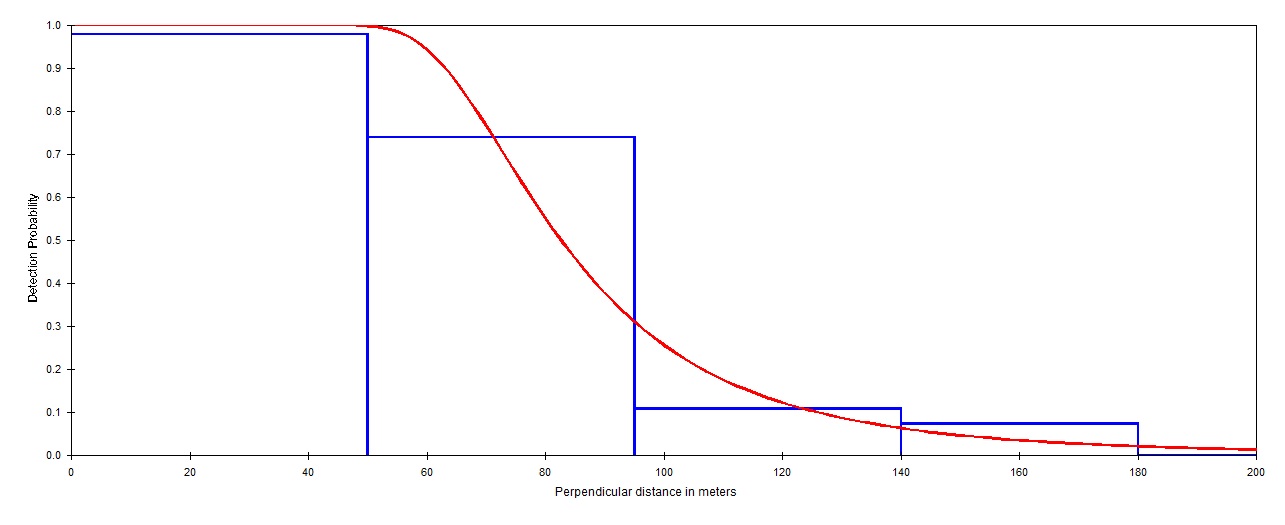


**Fig 35. Detection probability and distance for Wild pig data truncated at 200 m, and fitted with the hazard rate model in Coastal forests.**


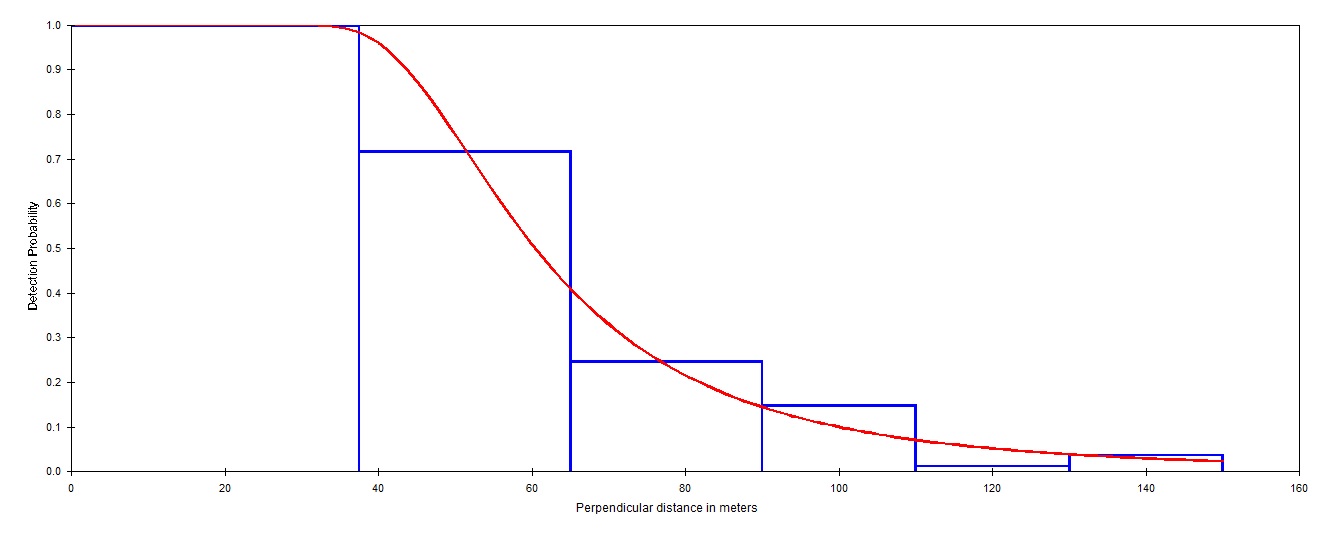


**Fig 36. Detection probability and distance data for Indian peafowl truncated at 150 m, and fitted with the hazard rate model in Coastal forests.**

*****
