# Supplementary material for: The semi-arid ecosystem of Asiatic Lion Landscape in Saurashtra, Gujarat: Population density, biomass and conservation of nine wild prey species
Source: PLoS One. 2023 Sep 28;18(9):e0292048. doi: 10.1371/journal.pone.0292048 (PMC10538734; doi:10.1371/journal.pone.0292048)
Supplement: S5 File — (DOCX) [file pone.0292048.s005.docx]

**Density estimates used in calculation of realized growth rate using regression.**

| **Study year** | **Reference** | **Species density** | | | |
| --- | --- | --- | --- | --- | --- |
|  |  | **Spotted deer** | **Sambar** | **Blue bull** | **Wild pig** |
| 1969 | Joslin 1974 | 4 | 0.24 | 0.85 | 0.23 |
| 1970 | Berwick | 3 | 0.47 | 0.51 | NA |
| 1987 | Khan 1993 | 53 | 3.1 | 0.51 | 0.65 |
| 1988 | Khan 1993 | 56 | 2.1 | 1.02 | 0.91 |
| 1989 | Khan 1993 | 48 | 0.93 | 0.36 | NA |
| 2002 | Khan et al., 2005 | 54 | 2.4 | NA | 1.6 |
| 2003 | Khan et al., 2005 | 47 | 1.7 | 0.5 | 1.36 |
| 2004 | Khan et al., 2005 | 41 | 2 | NA | NA |
| 2006 | Dave 2008 | 45 | 2.86 | 1.16 | 0.77 |
| 2009 | Zehra 2014 | 59 | 1.28 | 1.16 | 2.98 |
| 2010 | Zehra 2014 | 64 | 1.4 | 2.39 | 3.68 |
| 2011 | Zehra 2014 | 65 | 2.23 | 1.99 | 3.23 |
| 2015 | Gogoi et al., 2020 | 63 | 3.84 | NA | NA |
| 2017 | Chaudhary 2020 | 48 | 2 | 0.45 | 2 |
| 2019 | Ram et al. | 53 | 3.62 | 1.88 | 5.35 |
| 2022 | Present study | 58 | 3.99 | 0.66 | 2.21 |

NA=Density Not Assessed
