# Supplementary material for: The semi-arid ecosystem of Asiatic Lion Landscape in Saurashtra, Gujarat: Population density, biomass and conservation of nine wild prey species
Source: PLoS One. 2023 Sep 28;18(9):e0292048. doi: 10.1371/journal.pone.0292048 (PMC10538734; doi:10.1371/journal.pone.0292048)
Supplement: S6 File — (DOCX) [file pone.0292048.s006.docx]

**Detailed results of wild prey species at different study sites.**

**Gir**

Among the nine wild prey species surveyed in Gir, we found that spotted deer has got the highest density (per km^2^) (58.37±7.05 S.E.) followed by hanuman langur (41.62±8.87), Indian peafowl (37.01±5.05), sambar (3.99±0.71), wild pig (2.21±0.61), blue bull (0.65±0.18) and Indian gazelle (0.08±0.04) (Table 1). Due to the very low sample size, the four-horned antelope and blackbuck’s density was not estimated. Biomass estimation analysis found that spotted deer has got the highest biomass (kg) (3704355±447435) followed by sambar (900320±160160), hanuman langur (704364±150108), Indian peafowl (260975±35610), blue bull (165060±45720), wild pig (140265±38700) and Indian gazelle (2260±1120). Blue bull (136:100) and Indian gazelle (105:100) in Gir indicate sex ratio skewed towards male while the spotted deer (39:100), sambar (44:100), wild pig (63:100) and peafowl (82:100) sex ratio was skewed towards female. The fawn/piglet to adult female sex ratio was the highest for wild pig (60:100), followed by blue bull (20:100), sambar (20:100), spotted deer (18:100) and Indian gazelle (0.11:100).

Table 1. Density of wild prey species in Gir National Park and Wildlife Sanctuary.

| **Species** | **Model Name** | **D±SE** | **MGS±SE** | **GD±SE** | **GER (95% CI)** | **CV (%)** | **ESW±SE** |
| --- | --- | --- | --- | --- | --- | --- | --- |
| **Spotted**  **deer** | UN+COS | 58.37±7.05 | 7.45±0.21 | 7.82±0.01 | 0.61 (0.48-78) | 12 | 39.97±0.83 |
| **Sambar** | HR+COS | 3.99±0.71 | 2.57±0.11 | 1.54±0.26 | 0.14 (0.09-0.20) | 17 | 45.70±2.22 |
| **Blue bull** | HN+COS | 0.65±0.18 | 1.92±0.15 | 0.33±0.09 | 0.04 (0.02-0.07) | 29 | 63.44±6.33 |
| **Indian**  **gazelle** | HN+COS | 0.08±0.04 | 1.42±0.13 | 0.06±0.03 | 0.01 (0.004-0.03) | 57 | 113.76±20.71 |
| **Four-horned**  **antelope** | NA | 0.009 | NA | NA | 0.003 | NA | NA |
| **Blackbuck** | NA | NA | NA | NA | NA | NA | NA |
| **Wild pig** | HN+COS | 2.21±0.61 | 5.52±0.86 | 0.42±0.09 | 0.04 (0.02-0.06) | 27 | 48.03±4.75 |
| **Hanuman**  **langur** | HN+COS | 41.62±8.87 | 13.54±0.71 | 3.07±0.63 | 0.24 (0.16-0.35) | 21 | 39.11±3.52 |
| **Indian**  **peafowl** | HR+COS | 37.01±5.05 | 3.59±0.08 | 10.28±1.38 | 0.79 (0.61-1.03) | 13 | 38.79±2.03 |

UN=Uniform; HR=Hazard rare; HN=Half Normal; Cos=Cosine; D=Density; S.E.=Standard Error; MGS=Mean group size; GER=Group Encounter Rate; CI=Confidence Interval; CV=Coefficient of Variance; ESW=Effective Strip Width; NA=Not assessed due to lack of adequate sample size

**Mitiyala**

We got a sample size sufficient only for spotted deer, blue bull and Indian peafowl to carry out analysis, hence restricting the results to these species. The highest density in Mitiyala was of spotted deer (76.31±8.19), followed by Indian peafowl (48.58±5.23) and blue bull (2.40±0.28) (Table 2). The highest biomass was of wild pig (62550±6705), followed by blue bull (7920±900) and Indian peafowl (3695±475). Adult male to adult female ratio was skewed toward females in spotted deer (38:100), blue bull (45:100) and Indian peafowl (70:100). Fawn to adult female ratio in spotted deer was 8:100. At the same time, with no detection of fawn/peachicks, it was not calculated for blue bull and peafowl.

Table 2. Density of wild prey species in Mitiyala Wildlife Sanctuary.

| **Species** | **Model Name** | **D±SE** | **MGS±SE** | **GD±SE** | **GER (95% CI)** | **CV (%)** | **ESW±SE** |
| --- | --- | --- | --- | --- | --- | --- | --- |
| **Spotted**  **deer** | HR+COS | 76.31±8.19 | 7.45±0.21 | 6.46±0.39 | 0.84 (0.84-0.88) | 10 | 35.91±3.16 |
| **Sambar** | NA | 0.16* | NA | NA | 0.07 | NA | NA |
| **Blue bull** | UN+COS | 2.40±0.28 | 1.34±0.14 | 1.78±0.08 | 0.16 (0.16-0.16) | 10 | 45.00±2.15 |
| **Indian**  **gazelle** | NA | NA | NA | NA | NA | NA | NA |
| **Four-horned**  **antelope** | NA | NA | NA | NA | NA | NA | NA |
| **Blackbuck** | NA | NA | NA | NA | NA | NA | NA |
| **Wild pig** | NA | 0.60* | NA | NA | 0.14 | NA | NA |
| **Hanuman**  **langur** | NA | NA | NA | NA | NA | NA | NA |
| **Indian**  **peafowl** | HN+COS | 40.58±5.23 | 4.07±0.35 | 9.96±0.95 | 0.57 (0.57-0.57) | 12 | 28.67±2.74 |

**Paniya**

In Paniya, spotted deer and peafowl density was assessed as sufficient sample size was available for these species only. Spotted deer and Indian peafowl densities were 39.02±10.51 and 36.42±10.12, respectively. Similarly, the highest biomass was of spotted deer (69615±18765), followed by Indian peafowl (7220±2005) (Table 3). The adult male to adult female ratio was skewed towards females for spotted deer (63:100) and Indian peafowl (47:100). The fawn to adult female ratio in spotted deer was 26:100. At the same time, with no peachicks detected, it was not calculated for Indian peafowl.

Table 3. Density of wild prey species in Paniya Wildlife Sanctuary**.**

| **Species** | **Model Name** | **D±SE** | **MGS±SE** | **GD±SE** | **GER (95% CI)** | **CV (%)** | **ESW±SE** |
| --- | --- | --- | --- | --- | --- | --- | --- |
| **Spotted**  **deer** | HN+COS | 39.02±10.51 | 6.56 ±1.25 | 5.94±1.12 | 0.37 (0.20-0.66) | 26 | 31.20±4.11 |
| **Sambar** | NA | 0.15* | NA | NA | 0.04 | NA | NA |
| **Blue bull** | NA | 0.20* | NA | NA | 0.02 | NA | NA |
| **Indian**  **gazelle** | NA | 0.30* | NA | NA | 0.04 | NA | NA |
| **Four-horned**  **antelope** | NA | NA | NA | NA | NA | NA | NA |
| **Blackbuck** | NA | NA | NA | NA | NA | NA | NA |
| **Wild pig** | NA | 0.80* | NA | NA | 0.06 | NA | NA |
| **Hanuman**  **langur** | NA | 1.46* | NA | NA | 0.05 | NA | NA |
| **Indian**  **peafowl** | HN+COS | 36.42±10.12 | 4.15±0.48 | 8.77±2.21 | 0.58 (0.22-1.57) | 26 | 33.59±3.44 |

**Girnar**

In Girnar, the highest density was of Indian peafowl (68.87±25.39) followed by hanuman langur (46.00±27.00), spotted deer (31.18±10.80), sambar (11.54±1.95) and blue bull (1.12±0.49) (Table 4). The highest biomass was of sambar (330240±55840) followed by spotted deer (250965±86940), hanuman langur (98736±57948), Indian peafowl (61595±22710) and blue bull (36000±15840). Adult male to adult female sex was skewed towards females for all the wild prey species (Indian peafowl: 67:100; hanuman langur 38:100; spotted deer: 59:100; sambar 48:100 and blue bull: 53:100). Infant/fawn to adult female ratio was the highest for hanuman langur (45:100) followed by spotted deer (13:100), sambar (11:100) and blue bull (3:100).

Table 4**.** Density of wild prey species in Girnar Wildlife Sanctuary.

| **Species** | **Model Name** | **D±SE** | **MGS±SE** | **GD±SE** | **GER (95% CI)** | **CV (%)** | **ESW±SE** |
| --- | --- | --- | --- | --- | --- | --- | --- |
| **Spotted**  **deer** | HN+COS | 31.18±10.80 | 4.97±0.27 | 6.28±2.14 | 0.45  (0.16-1.28) | 34 | 36.22±2.17. |
| **Sambar** | HR+COS | 11.54±1.95 | 5.55±0.71 | 2.07 ±0.22 | 0.21  (0.17-0.24) | 16 | 50.71±4.92 |
| **Blue bull** | UN+COS | 1.12±0.49 | 3.17±0.53 | 0.35±0.14 | 0.04  (0.01-0.17) | 43 | 70.00±00 |
| **Indian**  **gazelle** | NA | NA | NA | NA | NA | NA | NA |
| **Four-horned**  **antelope** | NA | 0.01* | NA | NA | NA | 0.01 | NA |
| **Blackbuck** | NA | NA | NA | NA | NA | NA | NA |
| **Wild pig** | NA | 0.36* | NA | 0.02 | 0.005 |  | NA |
| **Hanuman**  **langur** | HN+COS | 46.00±27.00 | 17.86±1.97 | 2.57±1.53 | 0.10  (0.01-0.58) | 60 | 20.50±2.73 |
| **Indian**  **peafowl** | HN+COS | 68.87±25.39 | 7.21±0.53 | 9.68±3.44 | 0.75  (0.25-2.24) | 36 | 30.17±2.28 |

**Gir Grasslands**

In the Gir grasslands, the highest density was of spotted deer (46.94±11.40) followed by Indian peafowl (18.55±2.48), blue bull (9.66±2.73), wild pig (4.33±1.14), hanuman langur (3.27±1.32) and Indian gazelle (0.65±0.20) (Table 5). The highest biomass was of spotted deer (310275±75375) followed by blue bull (255600±72180), wild pig (28620±7560), Indian peafowl (13630±1820), hanuman langur (5772±2328) and Indian gazelle (1920±580). Adult male to adult female sex ratio was skewed towards females in spotted deer (32:100), blue bull (50:100), Indian gazelle (75:100), wild pig (83:100), and hanuman langur (40:100). For Indian peafowl male to female ratio was almost equal (101:100). Fawn/piglet/infant to adult female ratio was highest for wild pig (45:100) followed by hanuman langur (40:100), spotted deer (17:100), Indian gazelle (10:100) and blue bull (6:100).

Table 5. Density of wild prey species in Gir grasslands.

| **Species** | **Model Name** | **D±SE** | **MGS±SE** | **GD±SE** | **GER (95% CI)** | **CV (%)** | **ESW±SE** |
| --- | --- | --- | --- | --- | --- | --- | --- |
| **Spotted**  **deer** | HR+COS | 46.94±11.40 | 14.00±0.68 | 3.35±0.79 | 0.40  (0.25-0.65) | 24 | 60.57±2.77 |
| **Sambar** | NA | 0.15* | NA | NA | 0.06 | NA | NA |
| **Blue bull** | HN+COS | 9.66±2.73 | 5.83±0.37 | 1.65±0.45 | 0.49  (0.38-0.64) | 28 | 83.64±7.94 |
| **Indian**  **gazelle** | HR+COS | 0.65±0.20 | 2.51±0.19 | 0.26±0.07 | 0.05  (0.02-0.09) | 31 | 103.10±8.22 |
| **Four-horned**  **antelope** | NA | 0.02* | NA | NA | 0.03 | NA | NA |
| **Blackbuck** | NA | 0.24* | NA | NA | 0.05 | NA | NA |
| **Wild pig** | HR+COS | 4.33±1.14 | 8.97±1.31 | 0.48±0.10 | 0.06  (0.04-0.10) | 26 | 72.03±6.18 |
| **Hanuman**  **langur** | UN+COS | 3.27±1.32 | 17.23±4.16 | 0.19±0.06 | 0.02  (0.01-0.04) | 40 | 58.44±6.58 |
| **Indian**  **Peafowl** | HN+COS | 18.55±2.48 | 4.04±0.18 | 4.58±0.57 | 0.49  (0.38-0.64) | 13 | 54.36±1.86 |

**Junagadh Grasslands**

The highest density in Junagadh grasslands was of blue bull (16.58±5.60) followed by blackbuck (12.57±6.90), Indian peafowl (10.54±3.61), spotted deer (9.45±5.50) and wild pig (7.38±3.89) (Table 6). The highest biomass was of blue bull (151740±51300), followed by spotted deer (21600±12555), wild pig (16875±8910), blackbucks (12780±7020) and Indian peafowl (2680±915). The adult male to adult female sex ratio of wild prey species was skewed towards females (blue bull 37:100; spotted deer 29:100; blackbucks 38:100; wild pig 57:100) except Indian peafowl (111:100). Fawn/piglet to adult female ratio was the highest for wild pig (32:100) followed by blue bull (4:100), spotted deer (17:100), and blackbucks (16:100).

Table 6. Density of wild prey species in Junagadh grasslands.

| **Species** | **Model Name** | **D±SE** | **MGS±SE** | **GD±SE** | **GER (95% CI)** | **CV (%)** | **ESW±SE** |
| --- | --- | --- | --- | --- | --- | --- | --- |
| **Spotted**  **deer** | HR+COS | 9.45±5.50 | 7.95±0.75 | 1.25±0.71 | 0.22 (0.06-0.70) | 50% | 88.08±11.33 |
| **Sambar** | NA | NA | NA | NA | NA | NA | NA |
| **Blue bull** | HN+COS | 16.58±5.60 | 7.54±0.61 | 2.19±0.73 | 0.51 (0.25-1.05) | 31 | 117.01±6.18 |
| **Indian**  **gazelle** | NA | 0.35* | NA | NA | 0.15 | NA | NA |
| **Four-horned**  **antelope** | NA | NA | NA | NA | NA | NA | NA |
| **Blackbuck** | HN+COS | 12.57±6.90 | 18.15±3.60 | 0.69±0.35 | 0.11 (0.04-0.32) | 50 | 84.82±14.26 |
| **Wild pig** | HN+COS | 7.38±3.89 | 13.55±0.61 | 0.54±0.26 | 0.08 (0.03-0.21) | 51 | 73.71±12.70 |
| **Hanuman**  **langur** | NA | NA | NA | NA | NA | NA | NA |
| **Indian**  **peafowl** | HN+COS | 10.54±3.61 | 3.92±0.29 | 2.68±0.89 | 0.42 (0.21-0.87) | 31 | 79.87±4.93 |

**Bhavnagar Grasslands**

The highest density in grasslands of Bhavnagar was Indian peafowl (24.12±7.90) followed by blue bull (22.39±4.12), spotted deer (8.86±1.42), wild pig (2.66±1.41), and Indian gazelle (2.39±0.79) (Table 7). The highest biomass was of blue bull (365400±67320), followed by spotted deer (36135±5805), Indian peafowl (10935±3580), wild pig (10854±5760) and Indian gazelle (4340±1440). The adult male to adult female sex ratio of all the wild prey species was skewed towards female (blue bull 43:100; spotted deer 39:100; wild pig 70:100; Indian gazelle 66:100) except Indian peafowl (121:100). Fawn/piglet to adult female ratio was the highest in wild pig (174:100), followed by spotted deer (32:100), blue bull (10:100) and Indian gazelle (7:100).

Table 7. Density of wild prey species in Bhavnagar grasslands.

| **Species** | **Model Name** | **D±SE** | **MGS±SE** | **GD±SE** | **GER (95% CI)** | **CV (%)** | **ESW±SE** |
| --- | --- | --- | --- | --- | --- | --- | --- |
| **Spotted**  **deer** | HN+COS | 8.86±1.42 | 8.86±1.42 | 1.30±1.14 | 0.10 (0.02-0.50) | 89 | 39.55±4.06 |
| **Sambar** | NA | 0.28* | NA | NA | 0.07 | NA | NA |
| **Blue bull** | HR+COS | 22.39±4.12 | 6.52±0.37 | 3.43±0.60 | 0.73 (0.51-1.03) | 18 | 106.66±6.07 |
| **Indian**  **gazelle** | HR+COS | 2.39±0.79 | 2.26±0.11 | 1.05±0.34 | 0.29 (0.15-0.56) | 33 | 138.82±8.22 |
| **Four-horned**  **antelope** | NA | NA | NA | NA | NA | NA | NA |
| **Blackbuck** | NA | 4.87* | NA | NA | 0.10 | NA | NA |
| **Wild pig** | UN+COS | 2.66±1.41 | 0.24±0.08 | 10.83±4.42 | 0.03 (0.01-0.06) | 53 | 72.42±11.96 |
| **Hanuman**  **langur** | NA | NA | NA | NA | NA | NA | NA |
| **Indian**  **peafowl** | HN+COS | 24.12±7.90 | 4.34±0.24 | 5.95±1.79 | 0.70 (0.36-1.30) | 31 | 63.31±3.07 |

**Coastal forests**

In coastal forests, the highest density was of blue bull (21.52±2.59), followed by Indian peafowl (11.85±1.84), spotted deer (9.59±3.29) and wild pig (7.13±1.48) (Table 8). The highest biomass was of blue bull (213480±25740), followed by spotted deer (23805±8145) and wild pig (17685±3690) and Indian peafowl (3265±505). The adult male to adult female sex ratio for all the wild prey species was skewed towards females (blue bull 45:100; spotted deer 36:100; wild pig 74:100) except Indian peafowl (113:100). Fawn/piglet to adult female ratio was the highest in wild pig (89:100) followed by blue bull (14:100) and spotted deer (11:100).

Table 8. Density of wild prey species in Coastal forests.

| **Species** | **Model Name** | **D±SE** | **MGS±SE** | **GD±SE** | **GER (95% CI)** | **CV (%)** | **ESW±SE** |
| --- | --- | --- | --- | --- | --- | --- | --- |
| **Spotted**  **deer** | UN+COS | 9.59±3.29 | 13.37±1.51 | 0.71±0.23 | 0.10 (0.05-0.19) | 31 | 71.91±3.34 |
| **Sambar** | NA | NA | NA | NA | NA | NA | NA |
| **Blue bull** | HN+COS | 21.52±2.59 | 5.91±0.28 | 3.70±0.46 | 0.62 (0.52-0.75) | 13 | 84.60±7.24 |
| **Indian**  **gazelle** | NA | NA | NA | NA | NA | NA | NA |
| **Four-horned**  **antelope** | NA | NA | NA | NA | NA | NA | NA |
| **Blackbuck** | NA | NA | NA | NA | NA | NA | NA |
| **Wild pig** | HR+COS | 7.13±1.48 | 8.21±0.91 | 0.86±0.15 | 0.15 (0.11-0.21) | 20 | 90.03±8.14 |
| **Hanuman**  **langur** | NA | NA | NA | NA | NA | NA | NA |
| **Indian**  **peafowl** | HR+COS | 11.85±1.84 | 3.50±0.17 | 3.38±0.49 | 0.45 (0.34-0.60) | 15 | 67.20±3.60 |

*****
