# Supplementary figures and images for: The semi-arid ecosystem of Asiatic Lion Landscape in Saurashtra, Gujarat: Population density, biomass and conservation of nine wild prey species
Source: PLoS One. 2023 Sep 28;18(9):e0292048. doi: 10.1371/journal.pone.0292048 (PMC10538734; doi:10.1371/journal.pone.0292048)

**The increasing trend of average rainfall in Gir National Park and Wildlife Sanctuary.**


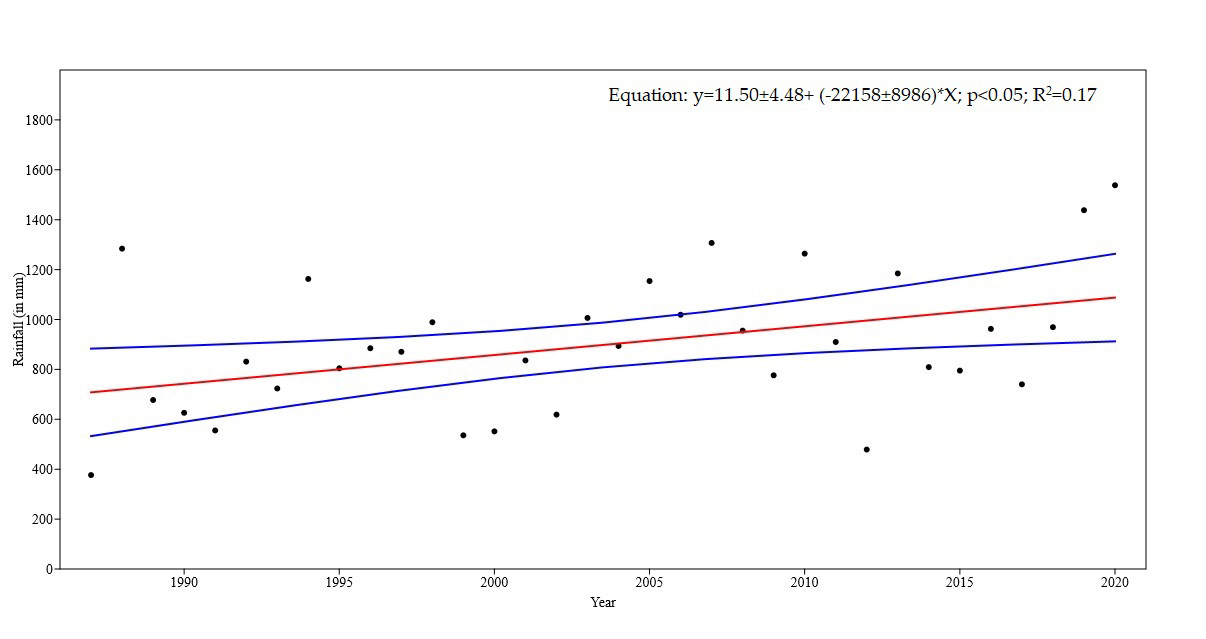


*****

Supplement: S7 File — (DOCX) [file pone.0292048.s007.docx]
